# Interferon‐Driven Biomarkers and Synergistic Therapy for PRMT5 Inhibition in Triple‐Negative Breast Cancer

**DOI:** 10.1002/advs.202505787

**Published:** 2025-11-19

**Authors:** Ziwen Zhang, Sheyu Zhang, Lu Guo, Yichun Pan, Juan Huang, Yishuai Ji, Jiaqi Tao, Yong Wei, Xiaojia Wang, Qin Wu

**Affiliations:** ^1^ Zhejiang Cancer Hospital Division of Breast Cancer Hangzhou Institute of Medicine (HIM) Chinese Academy of Sciences Hangzhou Zhejiang 310022 China; ^2^ School of Life Sciences Tianjin University Tianjin 300072 China; ^3^ Hangzhou Institute for Advanced Study University of Chinese Academy of Sciences Hangzhou 310024 China

**Keywords:** biomarker, DNA damage, interferon, PRMT5, TNBC

## Abstract

Triple‐negative breast cancer (TNBC) exhibits heterogeneous responses to PRMT5 inhibition, posing challenges for therapeutic targeting. Here, using PRMT5 inhibitors, coupled with transcriptomic profiling, basal interferon (IFN) signaling is identified as a biomarker of PRMT5 inhibition sensitivity. Sensitive TNBC models are characterized by elevated DNA damage, which correlated with enriched IFN pathway activity. Pharmacologically inducing DNA damage with the PARP inhibitor Olaparib activated IFN signaling and synergistically sensitized resistant TNBC cells to PRMT5 inhibition. Comprehensive pre‐clinical validation in patient‐derived organoid (PDO) and xenograft (PDX) models demonstrated robust antitumor efficacy of this combination therapy. Moreover, this dual targeting strategy reshaped the tumor microenvironment, enhancing dendritic cell‐CD8^+^ T cell crosstalk and conferring durable antitumor immunity in vivo. This study establishes IFN‐driven transcriptional signatures as predictive biomarkers for PRMT5 inhibitor response and unveils a rational combination strategy to overcome resistance in TNBC.

## Graphical abstract

Triple‐negative breast cancer exhibits variable sensitivity to PRMT5 inhibition. Basal interferon signaling is identified as a key biomarker of response. PARP inhibition with olaparib induces IFN signaling, sensitizing resistant TNBC cells to PRMT5 inhibitors. Combined treatment remodels the tumor microenvironment and enhances antitumor immunity, offering a precision strategy to overcome therapeutic resistance in TNBC.

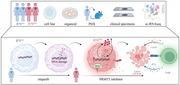

## 1 Introduction

Breast cancer remains the most prevalent malignancy and a leading cause of cancer‐related mortality among women worldwide.^[^ ^1^ ^]^ Triple‐negative breast cancer (TNBC), characterized by the absence of estrogen receptor, progesterone receptor, and human epidermal growth factor receptor 2 (HER2), presents a formidable challenge due to its limited responsiveness to traditional endocrine and HER2‐targeted therapies.^[^ ^2^ ^]^ Despite surgery and chemotherapy being the current mainstays of TNBC treatment,^[^ ^3^ ^]^ this aggressive subtype is notorious for its high rates of distant metastasis and local recurrence, which pose significant obstacles to effective clinical management.^[^ ^4^ ^]^ Therefore, understanding the pathogenesis of TNBC and identifying new therapeutic targets are crucial for enhancing the survival and quality of life of TNBC patients.

Protein arginine methyltransferases 5 (PRMT5), a type II PRMT, catalyzes symmetric dimethylation of arginine residues on histone and non‐histone proteins, regulating transcription, RNA splicing, and DNA damage responses.^[^ ^5^, ^6^, ^7^ ^]^ PRMT5 is frequently overexpressed in TNBC, promoting tumor proliferation, survival, and metastasis.^[^ ^8^ ^]^ PRMT5 inhibitors show promising safety and efficacy, particularly in cancers with MTAP deletion or splicing dysfunction.^[^ ^9^, ^10^, ^11^, ^12^ ^]^ However, these vulnerabilities are limited to specific cancer subtypes, and reliable biomarkers for PRMT5 inhibitor sensitivity in TNBC remain undefined.

In this study, we identify the activation of the interferon (IFN) pathway as a potential biomarker for sensitivity to PRMT5 inhibitors in TNBC. We also investigate the combination of PRMT5 inhibition with olaparib, a DNA‐damaging agent, as a strategy to overcome resistance and enhance therapeutic efficacy. This combination not only sensitizes TNBC cells to PRMT5 inhibition but also modulates the tumor microenvironment, stimulating anti‐tumor immune responses. Our findings offer insights into the clinical application of PRMT5 inhibitors and provide a more precise, personalized treatment strategy for TNBC patients.

## 2 Results

### 2.1 Heterogeneous Response to PRMT5 Inhibition in TNBC

To systematically assess the responsiveness of TNBC to PRMT5 inhibition, we first evaluated the sensitivity of 15 TNBC cell lines, representing various subtypes, including basal‐like (BL1 and BL2), mesenchymal (M), and mesenchymal stem‐like (MSL)^[^ ^2^ ^]^ (Table S1, Supporting Information). These cell lines were treated with two mechanistically distinct PRMT5 inhibitors, GSK591 and LLY‐283, along with their respective inactive analogs (LLY‐284, SGC2096) as negative controls (Figure S1a, Table S2, Supporting Information). GSK591 is a substrate‐competitive inhibitor that targets the PRMT5‐MEP50 complex,^[^ ^13^ ^]^ while LLY‐283 is a cofactor‐competitive inhibitor that binds to the S‐adenosylmethionine (SAM)‐binding site of PRMT5.^[^ ^14^ ^]^ Cells were cultured until untreated controls reached confluence, and the area above the curve (AAC) was calculated across nine incremental doses, ranging from 0 to 10 µm, to reflect the relative responsiveness of each cell line to both inhibitors (Figure S1b, Supporting Information).

**Figure 1:**
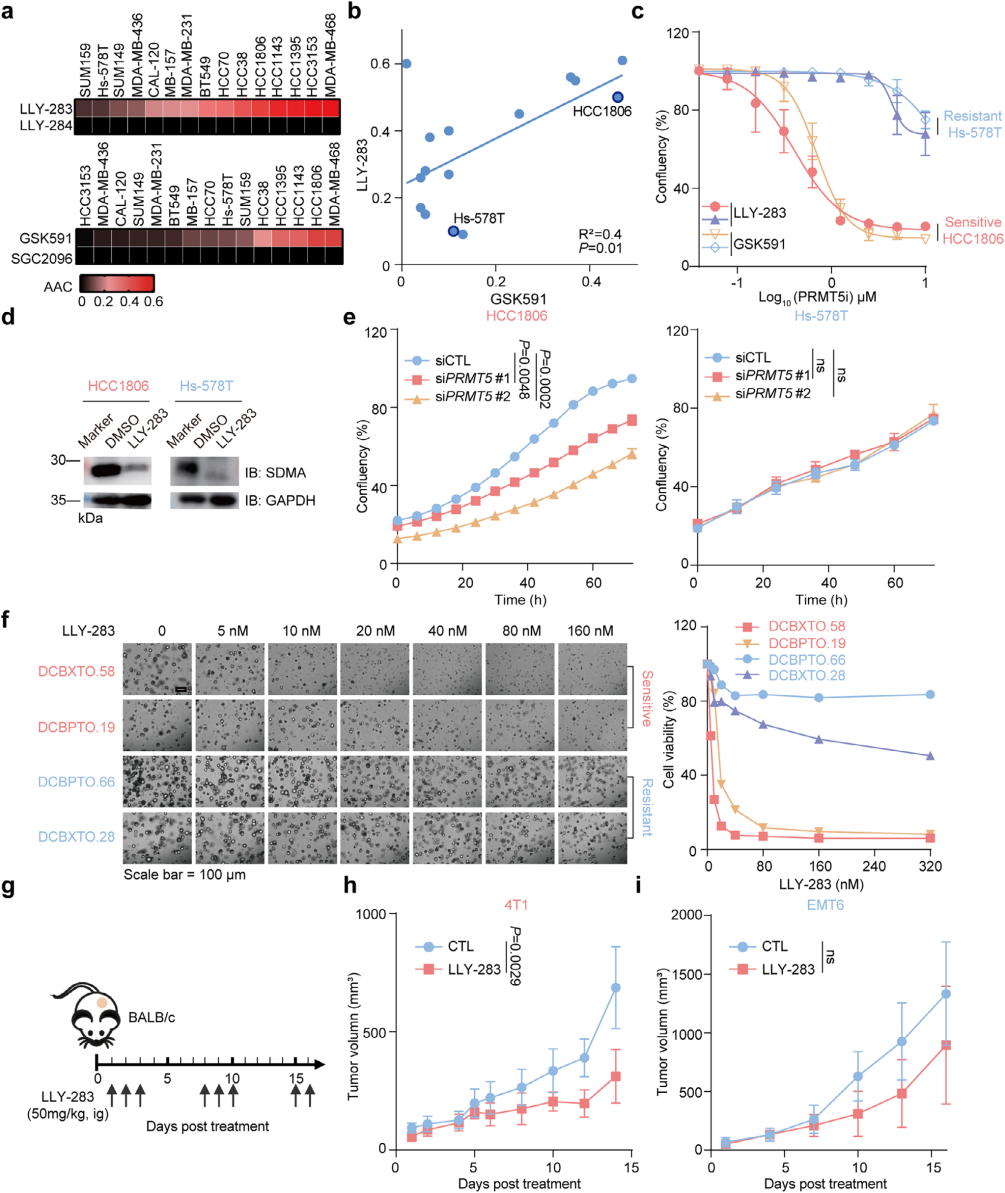
Heterogeneous response to PRMT5 inhibition in TNBC. a) Heat map of responsiveness to PRMT5 inhibitors in the indicated cell lines. The Area Above the Curve (AAC) was calculated from dose–response assays across 15 TNBC cell lines. Data are normalized to DMSO. A higher AAC indicates greater sensitivity. *n* = 4. b) Correlation analysis of AAC values across different TNBC cell lines treated with LLY‐283 and GSK591. c) Growth curves of HCC1806 and Hs‐578T cells treated with LLY‐283 or GSK591 for 5 days at indicated concentrations. Data are shown as mean ± s.d., *n* = 4. d) Immunoblots of SDMA of HCC1806 and Hs‐578T cells following treatment of LLY‐283 (1 µm, 5 days) or DMSO. Data are representative of independent experiments. e) Growth curves of siRNA knockdown of *PRMT5* or control in HCC1806 (left panel) and Hs‐578T (right panel) cells. Data are shown as mean ± s.d., *n* = 4, two‐sided unpaired *t*‐test. f) Left panel: representative images of organoid models exposed to DMSO or indicated concentrations of LLY‐283, scale bar, 100 µm. Right panel: differential response of organoids to LLY‐283 treatment. Data are shown as mean ± s.d., *n* = 3. g) Dose schedule. h) Tumor growing curves of 4T1 tumors in immunocompetent BALB/c mice. Data are shown as mean ± s.d., *n* = 9, two‐sided unpaired *t*‐test. i) Tumor growing curves of EMT6 tumors in immunocompetent BALB/c mice. Data are shown as mean ± s.d., *n* = 8, two‐sided unpaired *t*‐test. ns represents not significant.

As expected, we observed differential responses of TNBC cells to both PRMT5 inhibitors. A subset of TNBC cells showed sensitivity, reflected by an AAC higher than 0.2. Conversely, other cell lines did not respond to PRMT5 inhibition (**Figures** **1**a; S1c, Supporting Information). While the varied AAC values indicated the heterogeneous responses to PRMT5 inhibitors, we observed similar inhibitory patterns between the two PRMT5 inhibitors, as reflected by a strong correlation of AAC values for LLY‐283 and GSK591 (Figure 1b). It is known that PRMT5 is responsible for the majority of the symmetric dimethylation of arginine (SDMA) in cells. To confirm that PRMT5 inhibitors were effective in inhibiting PRMT5 activity, we treated representative responder TNBC cell line (HCC1806) and non‐responder cell line (Hs‐578T) with 1 µm GSK591 or LLY‐283 for 5 days and measured the levels of SDMA. A strong reduction of the SDMA mark was observed in both responder and non‐responder cells, validating the on‐target effect of both PRMT5 inhibitors (Figure 1c,d).

Consistent with the results from pharmacological inhibition, PRMT5 silencing reduced PRMT5 protein levels and significantly impaired the growth of TNBC cell lines sensitive to PRMT5 inhibitor (HCC1806) but had no impact on the growth of PRMT5 inhibitor resistant cells (Hs‐578T) (Figures 1e; S1d, Supporting Information). These results suggest a shared mechanism underlying sensitivity to PRMT5 inhibition with either pharmacological or genetic inhibition.

To further evaluate the clinical relevance of this heterogeneous response, we assessed PRMT5 inhibitor sensitivity in patient‐derived xenograft organoids (PDXDOs), which are known to better recapitulate breast histology and epithelial heterogeneity, making them more suitable for evaluating drug responses in cancer therapy.^[^ ^15^ ^]^ PDXDOs derived from four different TNBC patients were cultured, and their sensitivity to PRMT5 inhibitors was tested. Consistent with our findings in TNBC cell lines, some PDXDOs were sensitive to PRMT5 inhibition, exhibiting IC_50_ values of less than 1 nm, while others were resistant, with IC_50_ values exceeding 320 nm (Figure 1f). Similarly, this differential response to PRMT5 inhibition was validated in mouse‐derived cell lines, where the PRMT5 inhibitor LLY‐283 significantly inhibited the proliferation of 4T1 cells while the effect on EMT6 cells was less pronounced (Figure S1e, Supporting Information). In vivo, mice bearing 4T1 xenografts treated with LLY‐283 on a 3‐days‐on/4‐days‐off schedule (as per previous studies^[^ ^16^ ^]^) exhibited significant tumor growth suppression without weight loss, whereas EMT6 tumors remained refractory under the same regimen(Figures 1g–i; S1f,g, Supporting Information). These results further confirm the high heterogeneity of TNBC and suggest that sensitivity to PRMT5 inhibition may be dependent on the molecular characteristics of the tumor cells.

### 2.2 Basal Interferon Response Correlates with TNBC Sensitivity to PRMT5 Inhibition

**Figure 2:**
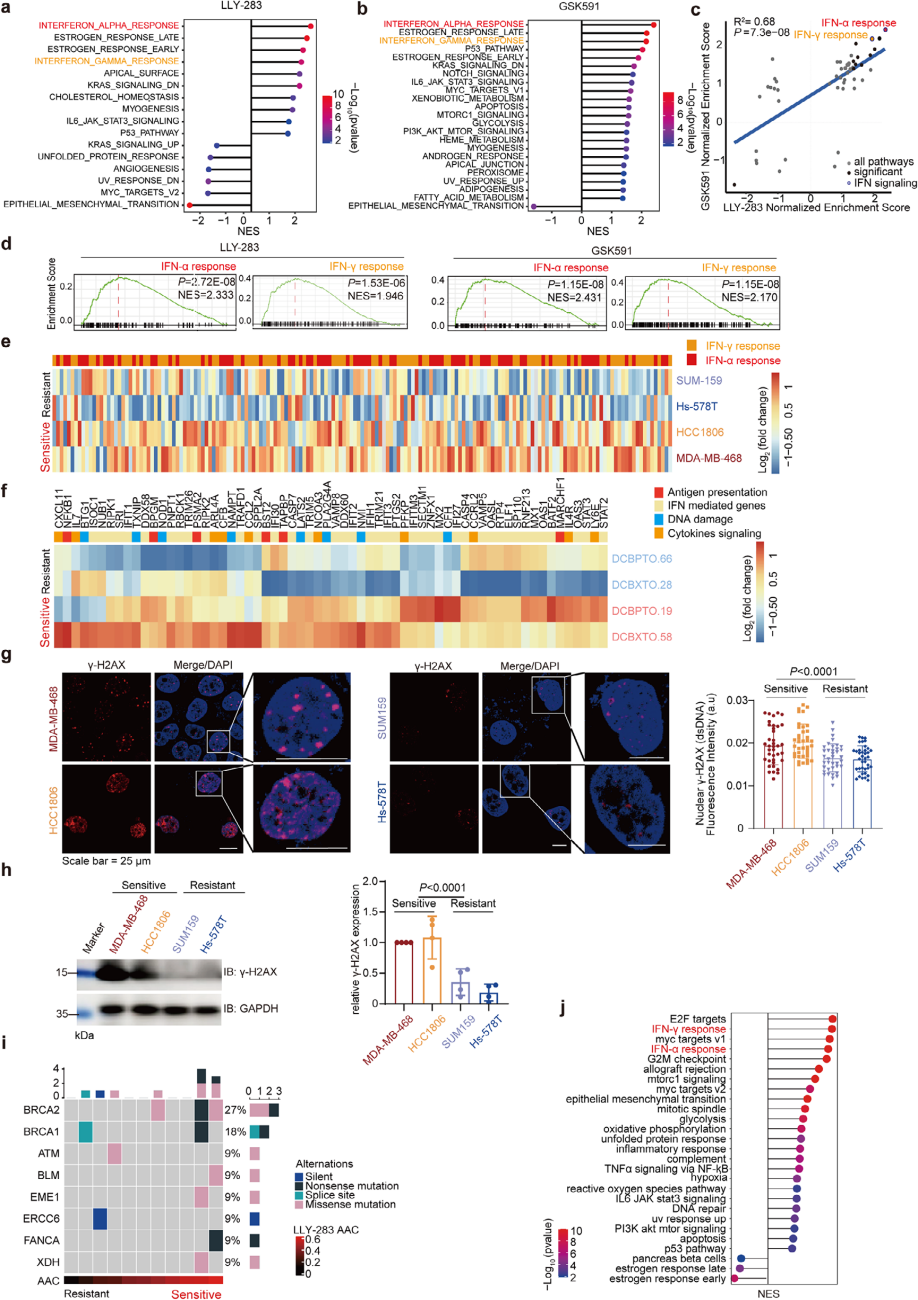
Basal interferon response correlates with TNBC sensitivity to PRMT5 inhibition. a, b) Hallmark pathway enrichment analysis of the differentially expressed genes (DEGs) associated with sensitivity to LLY‐283 (a) and GSK591 (b). c) Correlation analysis of the Normalized Enrichment Score (NES) value of HALLMARK pathways across different TNBC cell lines. d) GSEA plots of IFN‐α and IFN‐γ pathway. e) Heatmap of DEGs in the IFN‐α and IFN‐γ pathway between PRMT5i‐sensitive and PRMT5i‐resistant cell lines. f) Heatmap of differentially expressed IFN‐responsive genes between PRMT5i‐sensitive and PRMT5i‐resistant organoids. g) Immunofluorescence staining analysis of γ‐H2AX protein expression in PRMT5i‐sensitive (MDA‐MB‐468 and HCC1806) and PRMT5i‐insensitive (Hs‐578T and SUM159) TNBC cells. Left: representative micrographs; right: quantification of γ‐H2AX foci (n = 3 independent experiments, ≥85 cells per group). Scale bar, 25 µm. Data are shown as mean ± s.d., two‐sided unpaired *t*‐test. h) Western blot of γ‐H2AX protein expression in PRMT5i‐sensitive and insensitive cells. Left: representative micrographs; right: quantification of γ‐H2AX band intensities. Data are shown as mean ± s.d., *n* = 4, two‐sided unpaired *t*‐test. i) Oncoplot showing the gene mutations involved in DNA repair across TNBC cell lines. Data obtained from Cancer Cell Line Encyclopedia (CCLE). j) Hallmark pathways significantly correlated with *BRCA1/2* mutation in TNBC from TCGA datasets. One‐tailed Fisher's exact test.

The varied response of TNBC to PRMT5 inhibition prompted us to investigate the underlying mechanism. Initially, we assessed whether previously identified biomarkers of PRMT5i sensitivity‐such as *MTAP* deletions in AML^[^ ^17^, ^18^ ^]^ and the splicing dysfunction ratio of *CLNS1A*/*RIOK1* in glioblastoma^[^ ^16^ ^]^‐correlated with sensitivity in TNBC cell lines. However, neither marker showed a significant correlation with PRMT5 inhibitor sensitivity (Figure S2a–d, Supporting Information). We then employed an unbiased approach by comparing the responses of 15 TNBC cell lines to PRMT5 inhibitors, identifying gene expression patterns associated with sensitivity or resistance. We calculated the Pearson correlation coefficients between the AAC values for two PRMT5 inhibitors and the baseline expression levels of individual genes (Figure S2e, Supporting Information). These rank‐ordered gene lists were subsequently subjected to GSEA (**Figure** **2**a,b). Our analysis revealed a strong correlation in the gene signatures associated with PRMT5 activity between the two inhibitors (Figure 2c). Notably, the top two pathways within these enriched gene sets were related to IFNα and IFNγ signaling, with the corresponding genes visualized in the heatmap (Figures 2d,e; S2f, Supporting Information).

To quantify IFN pathway activation across models, we used the MSigDB “Interferon Alpha Response” and “Interferon Gamma Response” gene sets as a non‐redundant interferon signature (Table S3, Supporting Information) and applied single‐sample GSEA (ssGSEA). Normalized interferon signature scores varied markedly across cell lines, and high scores precisely tracked PRMT5i sensitivity (Figure S2f, Supporting Information). Consistent with these findings in cell lines, a comparison of the baseline transcriptomic data from TNBC organoids further confirmed the association between interferon‐related genes and sensitivity to PRMT5 inhibition. In particular, PRMT5i‐sensitive organoids (DCBXTO.58 and DCBXTO.19) exhibited enriched interferon signatures, while PRMT5i‐insensitive organoids (DCBPTO.66 and DCBXTO.28) did not (Figure 2f). Among the top upregulated genes in sensitive models, we observed increased expression of genes such as IFN‐responsive genes of *MX1* and *IFI30*, antigen presentation genes of *TAPBP* and *TRIM21*, cytokine signaling related genes of *CXCL11* and *IL7* and DNA damage related genes of *TXNIP* and *PLA2G4A* (Table S4, Supporting Information). Altogether, these results suggest that a preexisting enhanced expression of genes involved in the IFN signaling pathway might predict the responsiveness to PRMT5 inhibitors (Figure S2g, Table S5, Supporting Information).

We next tested the functional necessity of IFN signaling for PRMT5 inhibitor efficacy. siRNA‐mediated knockdown of *STAT1* or *IRF9* in the sensitive MDA‐MB‐468 cells abrogated PRMT5i sensitivity (Figure S3a–d, Supporting Information), confirming that intact IFN transcriptional machinery is required. Conversely, pretreatment of resistant SUM159 and Hs‐578T cells with recombinant IFN‐α or IFN‐β reinstated PRMT5i sensitivity (Figure S3e,f, Supporting Information), demonstrating that IFN pathway activation is sufficient to confer responsiveness.

Finally, we sought to explore the origins of the varied IFN activation levels across TNBC cells. Given that intracellular DNA damage‐induced dsDNA is a major driver of innate immune activation within cells,^[^ ^19^ ^]^ we quantified DNA damage and cytosolic dsDNA accumulation in PRMT5i‐sensitive and insensitive cells using western blotting and immunofluorescence assays. These analyses unveiled heightened accumulation of DNA damage and cytosolic dsDNA formation in PRMT5i‐sensitive cells (Figure 2g,h). Furthermore, mutation analysis uncovered a greater prevalence of gene mutations involved in DNA repair pathways, such as *BRCA1* and *BRCA2*, in PRMT5i‐sensitive cells (Figure 2i; Table S7, Supporting Information). This correlation between *BRCA* mutations and IFN activation was further validated in TNBC patient samples (Figure 2j). These findings suggest that disparities in baseline cellular DNA damage levels account for divergent intrinsic IFN activity.

Mechanistic links between DNA repair capacity and PRMT5i sensitivity were further substantiated in proteomic profiling of resistant cell lines, which identified six upregulated proteins—four of which (RAD50, ATM, pAMPK, and pAKT/pPKC) are directly or indirectly associated with the DNA damage response (DDR) (Figure S4a, Supporting Information).^[^ ^20^, ^21^ ^]^ RNA‐seq confirmed a strong correlation between RAD50 expression and PRMT5i resistance (R^2^ = 0.49, *P* = 0.0021) (Figure S4b, Supporting Information), with resistant lines consistently exhibiting higher RAD50 levels (Figure S4c, Supporting Information). To functionally validate the role of DDR reactivation in resistance, we treated PRMT5i‐resistant TNBC cells (Hs‐578T and MDA‐MB‐436) with DDR inhibitors targeting ATM (AZD0156^[^ ^22^ ^]^) or WEE1 (AZD1775^[^ ^23^ ^]^). Co‐treatment significantly enhanced PRMT5i sensitivity and reversed resistance (Figure S4d–g, Supporting Information), confirming DDR signaling as a functional resistance mechanism.

### 2.3 IFN‐Induced Splicing Alterations Contribute to PRMT5 Dependency

**Figure 3:**
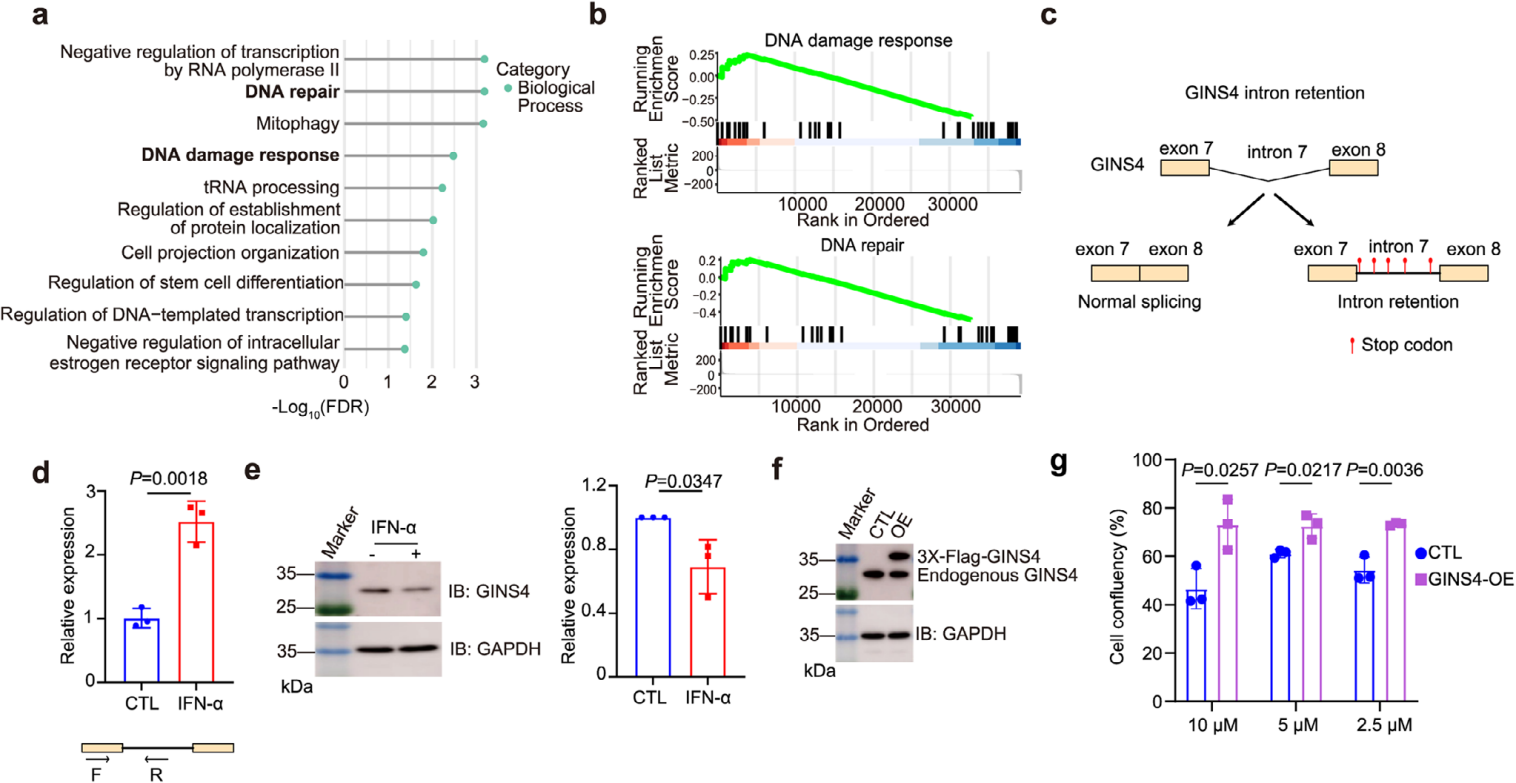
IFN‐induced splicing alterations contribute to PRMT5 dependency. a) GO Biological Process pathway enrichment analysis of the transcripts exhibiting AS events after interferon alpha treatment. b) GSEA plot of DNA damage response (up) and DNA repair (down) pathways. c) Schematic of *GINS4* intron retention following interferon‐alpha (IFN‐α) treatment. Intron retention induces a frameshift, leading to premature translational termination. The red bar denotes the stop codon. d) qPCR analysis of *GINS4* intron retention in SUM159 cells following IFN‐α treatment. Data are presented as mean ± s.d., *n* = 3, two‐sided unpaired *t*‐test. e) Representative immunoblots of GINS4 expression and loading control GAPDH in IFN‐α‐treated SUM159 cells (Left). Quantification of GINS4 band intensities (Right). Data are shown as mean ± s.d., *n* = 3, two‐sided unpaired *t*‐test. f) Representative immunoblots of GINS4 in SUM159 cells transfected with control (CTL) or GINS4 overexpression plasmids. g) Cell confluency of GINS4‐overexpressing SUM159 cells in response to co‐treatment with IFN‐α and indicate concentrations of LLY‐283. Data are presented as mean ± s.d., *n* = 3, two‐sided unpaired *t*‐test.

To explain how interferon signaling augments PRMT5 dependency, we performed RNA‐seq on cells with or without IFN‐α pre‐treatment. Interferon exposure induced widespread alternative splicing changes (Figure S4h, Supporting Information), particularly in genes governing DNA repair and damage response (Table S6, Supporting Information). And GSEA revealed that these aberrantly spliced transcripts were enriched in DNA repair and damage‐response pathways (**Figure** **3**a,b), suggesting that IFN‐driven splicing reprogramming compromises genome integrity and increases cellular dependence on PRMT5 for maintaining splicing fidelity.

To directly test whether these splicing defects contribute to PRMT5 dependency, we focused on *GINS4* as a representative target, which exhibits one of the most pronounced splicing alterations, specifically intron retention, which introduces a premature termination codon and disrupts gene function (Figure 3c). Moreover, GINS4 plays a critical role in DNA replication fork progression, directly linking it to genome stability.^[^ ^24^, ^25^, ^26^ ^]^ Indeed, IFN‐α treatment induced intron retention in *GINS4* (Figure 3d), leading to a marked reduction in protein expression (Figure 3e). Rescue experiments with splice‐corrected *GINS4* cDNA significantly alleviated the IFN‐α‐induced sensitivity to PRMT5 inhibition (Figure 3f,g). This result provides direct causal evidence that IFN‐induced splicing disruption of a DNA repair gene is sufficient to confer PRMT5 dependency.

Although other mis‐spliced genes involved in DNA repair likely contribute to the overall phenotype, our functional validation of *GINS4* establishes a mechanistic model in which IFN‐induced intron retention impairs DNA repair function, creating a therapeutic vulnerability to PRMT5 inhibition. These results suggest that interferon‐driven splicing reprogramming increases replication stress and DNA damage, thereby heightening reliance on PRMT5‐mediated symmetric arginine methylation of core splicing factors (e.g., Sm proteins, SRSFs) to preserve splicing fidelity and genome integrity.^[^ ^27^ ^]^

Together, our data support a model in which elevated baseline DNA damage in a subset of TNBCs drives intrinsic interferon signaling, which in turn promotes widespread splicing perturbations that amplify dependence on PRMT5 to maintain splicing fidelity and genome stability. This feed‐forward interplay between DNA damage, cGAS‐STING/IFN signaling, and PRMT5 dependency provides a mechanistic rationale for combining PRMT5 inhibitors with DNA‐damaging agents in TNBC. Further work will be required to quantify the relative contributions of additional mis‐spliced targets to this vulnerability and to translate these signatures into clinical patient stratification strategies.

### 2.4 Synergistic Sensitization of TNBC to PRMT5 Inhibition by Olaparib

**Figure 4:**
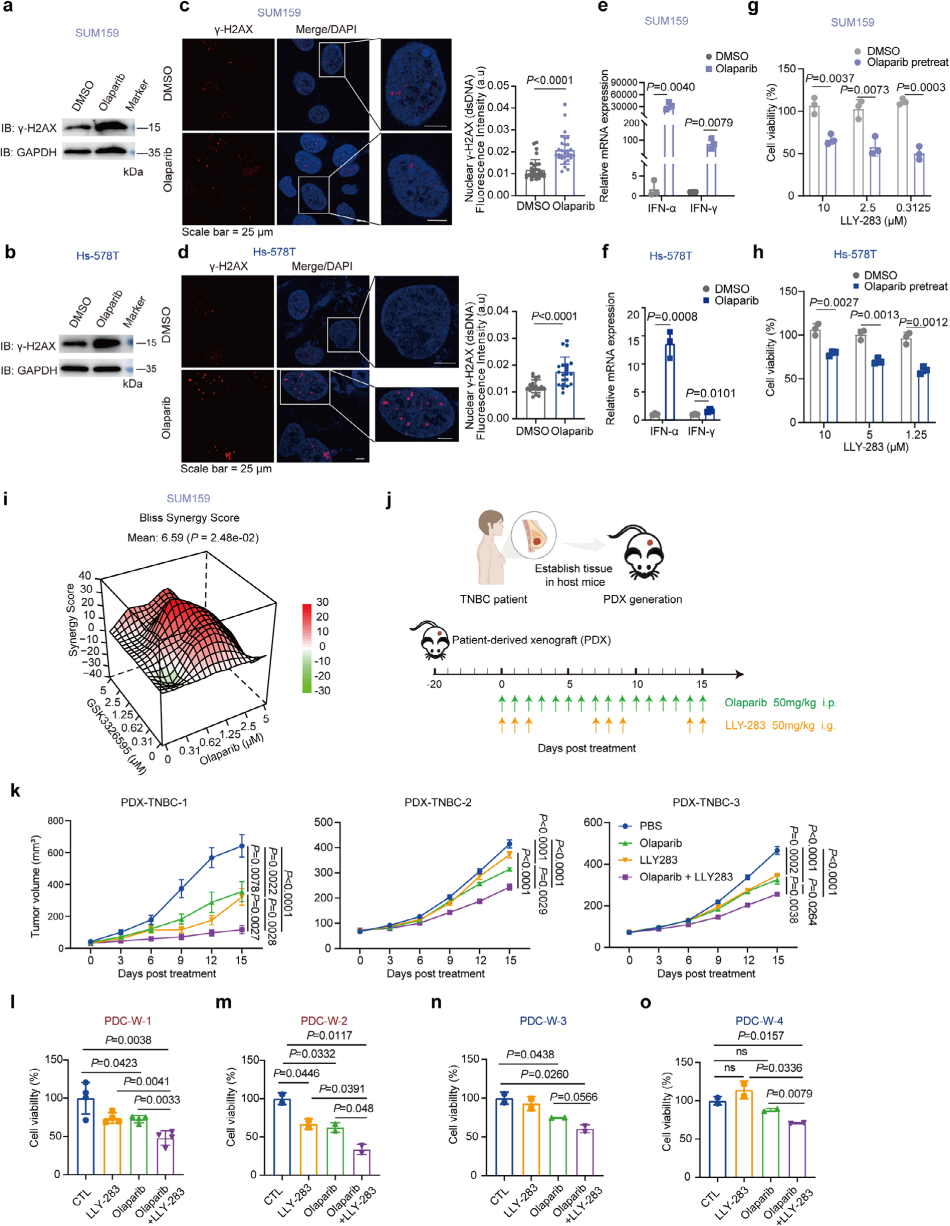
Synergistic Sensitization of TNBC to PRMT5 Inhibition by Olaparib. a–d) SUM159 and Hs‐578T cells were treated with DMSO or Olaparib (3 µm, 3 days). Western blot analysis (a, b) and immunofluorescence staining (c, d) were performed to determine γ‐H2AX expression. Scale bar, 25 µm. Independent biological experiments of at least 85 cells per group were analyzed. Data are shown as mean ± s.d., *n* = 3, two‐sided unpaired *t*‐test. e, f) qRT‐PCR was performed to determine IFN expression. Data are shown as mean ± s.d., *n* = 3, two‐sided unpaired *t*‐test. g, h) Cells were pre‐treated with Olaparib (3 µm, 3 days), followed by LLY‐283 treatment at indicated concentrations for 5 days. Cell viability was detected. Data are shown as mean ± s.d., *n* = 3, two‐sided unpaired *t*‐test. i) Bliss synergy score of GSK3326595 and Olaparib in SUM159. *n* = 6, two‐sided unpaired *t*‐test. j) Dose schedule. k) Tumor volume was measured in patient‐derived xenograft models that were administered PBS (CTL), LLY‐283 (50 mg kg^−1^, 3‐day‐on and 4‐day‐off), Olaparib (50 mg kg^−1^, every day), or LLY‐283 + Olaparib. Data are shown as mean ± sem, *n* = 9 (left), *n* = 6 (middle), *n* = 5 (right), one‐way ANOVA with Dunnett's test for multiple comparisons. l–o) Cell viability of patient‐derived cell (PDC) models was treated with LLY‐283, Olaparib, or their combination. All data were shown as mean ± s.d., two‐tailed Student's *t*‐test.

To confirm the hypothesis that induction of DNA damage can activate the IFN pathway and ultimately enhance sensitivity to PRMT5 inhibitors, we treated PRMT5 inhibitor‐resistant cell lines, SUM159 and Hs‐578T, with Olaparib, an FDA‐approved drug for breast cancer treatment known to induce DNA damage.^[^ ^28^ ^]^ As expected, Olaparib treatment led to elevated intracellular DNA damage (**Figure** **4**a,b) and dsDNA accumulation (Figure 4c,d). Consequent activation of the cGAS–STING pathway was evidenced by elevated STING levels and phosphorylation of IRF3 and TBK1 (Figure S5a, Supporting Information) along with a significant upregulation of IFN levels (Figure 4e,f), suggesting the activation of an immune response driven by the genomic instability induced by the treatment. Critically, siRNA knockdown of *STING* prevented IFN induction and restored PRMT5i resistance (Figure S5b,c, Supporting Information), establishing cGAS‐STING‐mediated innate immune sensing as the mechanistic bridge between DNA damage and PRMT5 inhibitor sensitivity.

We then investigated whether Olaparib could sensitize cells to PRMT5 inhibition. Indeed, cells pre‐treated with Olaparib exhibited higher sensitivity to PRMT5 inhibitors in both SUM159 and Hs‐578T cell lines (Figures 4g,h; S5d,e, Supporting Information). This synergistic effect was also observed in two additional cell lines (MDA‐MB‐436 and SUM149) and further validated using GSK3326595, a PRMT5 inhibitor currently undergoing clinical trials. (Figures 4i; S5f–j, Supporting Information). Together, these results underscore the translational potential of combining PARP and PRMT5 inhibition to overcome therapeutic resistance in TNBC.

Furthermore, we validated this synergistic effect between Olaparib and PRMT5 inhibition in TNBC patient‐derived xenograft (PDX) models. These three PDX models originated from distinct clinical contexts: PDX‐TNBC‐1 from a patient resistant to neoadjuvant AC‐T chemotherapy (doxorubicin/cyclophosphamide followed by paclitaxel); PDX‐TNBC‐2 was derived from an untreated patient; and PDX‐TNBC‐3 from a patient who received paclitaxel liposome plus cisplatin followed by gemcitabine and docetaxel (detailed in Table S8, Supporting Information). Despite these heterogeneous clinical backgrounds, and consistent with our in vitro findings, co‐treatment with Olaparib and PRMT5 inhibitors produced significantly greater tumor growth suppression than either agent alone (Figures 4j,k; S5k–n, Supporting Information). Collectively, these findings demonstrate that Olaparib sensitizes TNBC cells to PRMT5 inhibition by inducing DNA damage and activating the interferon signaling pathway, providing a compelling rationale for combining DNA‐damaging agents with PRMT5 inhibitors in the treatment of TNBC.

To further confirm the translational potential of this combination, we established four patient‐derived cell (PDC) models from fresh specimens. These PDC lines recapitulate the heterogeneity of the originating tumors, and preliminary sensitivity assays indicate that olaparib pre‐treatment similarly enhances PRMT5 inhibitor responses across multiple independent patient models (Figure 4l–o).

### 2.5 Olaparib Enhances Sensitivity of PRMT5 Inhibitors to Immunotherapy

**Figure 5:**
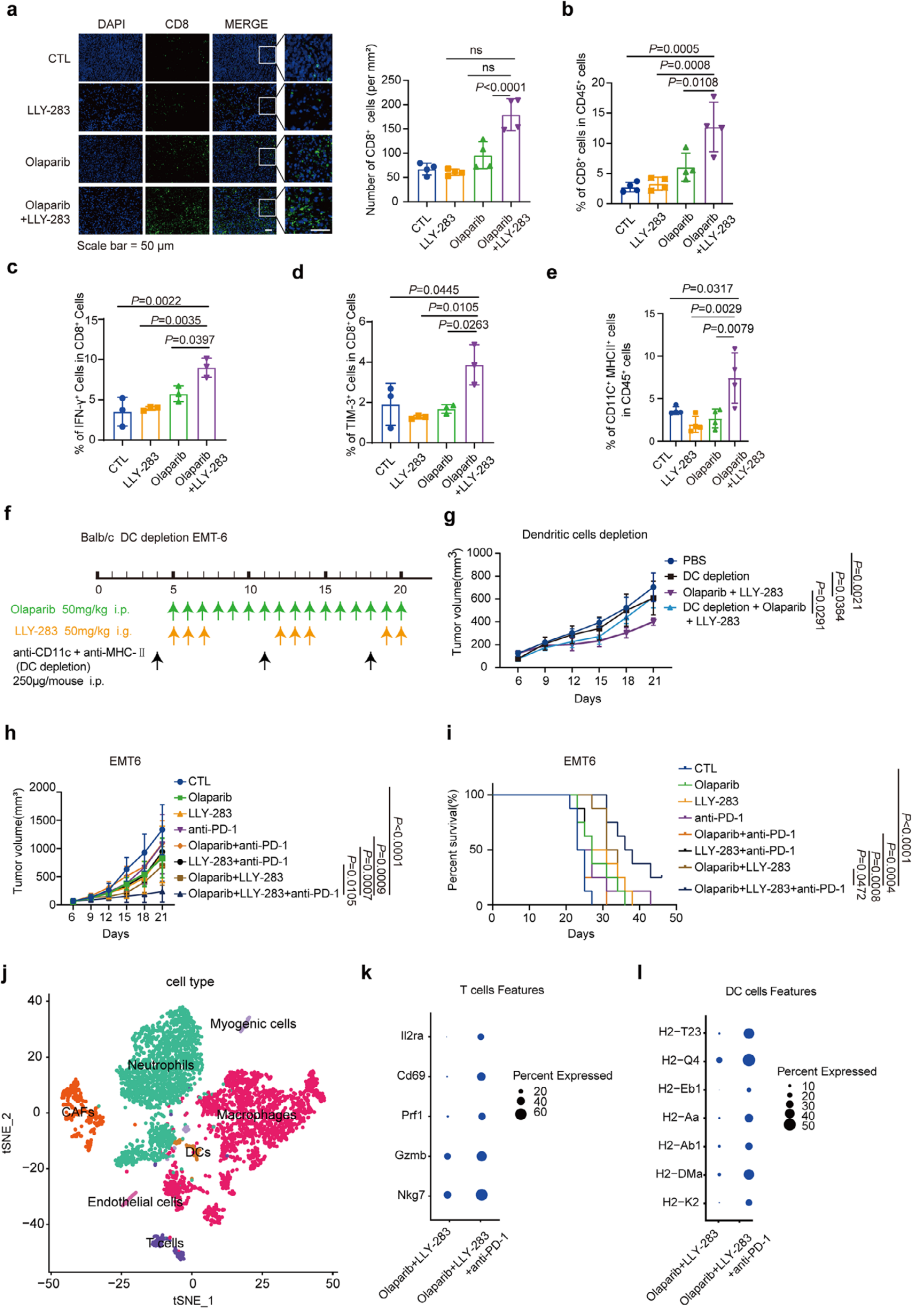
Olaparib enhances the sensitivity of PRMT5 inhibitors to immunotherapy. a) BALB/c mice bearing EMT6 tumors were treated with LLY‐283 (50 mg kg^−1^, 3‐day on and 4‐day off) and Olaparib (50 mg kg^−1^, every day), alone or in combination. Immunofluorescence staining was performed to detect tumor‐infiltrating CD8+ T cells. Scale bar, 50 µm. Data are shown as mean ± s.d., *n* = 4, one‐way ANOVA with Dunnett's test for multiple comparisons. b–e) Flow cytometry analysis was conducted to measure the proportion of CD8+ T cells within the CD45+ cells (b), IFN‐γ+ cells in CD8+ cells (c), TIM‐3+ cells in CD8+ cells (d), and the proportion of CD11C+ cells within the CD45+ proportion (e). Data are shown as mean ± s.d., *n* = 4, one‐way ANOVA with Dunnett's test for multiple comparisons. f) Schematic of the dendritic cell (DC) depletion schedule in the EMT6 syngeneic breast cancer model. g) Tumor volume trajectories for mice treated with PBS (blue, *n* = 5), DC depletion (red, *n* = 5), DC depletion combined with Olaparib + LLY‐283 (green, *n* = 5), or Olaparib + LLY‐283 (purple, *n* = 5). Data are shown as mean ± s.d. The statistical significance of differences at day 21 was evaluated using the one‐way ANOVA. h, i) Tumor growth curves (h) and Kaplan–Meier survival analysis (i) of mice bearing EMT6 tumors receiving the indicated treatments. Data are shown as mean ± s.d., *n* = 8 or 10 mice per group, one‐way ANOVA with Dunnett's test. j) t‐SNE plot of non‐tumor cell clusters of BALB/c mice bearing EMT6 tumors. k) Dot plot showing the expression of T cell cytotoxity markers (*Nkg7, Gzmb*, and *Prf1*) and activation markers (*Cd69* and *Il2ra*) across treatment groups. l) Dot plot showing the expression of major histocompatibility complex genes in DC cells.

As the combination of Olaparib and PRMT5 inhibitor displayed a potent IFN response, we embarked on investigating its potential to modulate the tumor microenvironment (TME) for optimal immune responses. We first examined EMT6 tumors post‐treatment with Olaparib, PRMT5 inhibitor, or the combination to profile immune cell populations. Immunofluorescence staining revealed a significant increase in tumor infiltrating CD8^+^ T cells in the combination treatment group compared to the monotherapy groups (**Figure** **5**a). This effect was further confirmed by the flow cytometry analysis (Figures 5b; S6a, Supporting Information). Moreover, these infiltrating CD8^+^T cells exhibited elevated IFN‐γ production alongside upregulated TIM‐3 expression, consistent with an activated yet regulated phenotype primed for checkpoint blockade (Figure 5c,d).

Effective anti‐tumor immune responses often rely on the cooperation between dendritic cells (DCs) and T cells, emphasizing the pivotal roles of CD11^+^ DCs in the anti‐tumor T cell response.^[^ ^29^, ^30^ ^]^ In line with this, mice treated with the combination of Olaparib and PRMT5 inhibitor exhibited increased populations of CD45^+^CD11^+^MHCII^+^ cells compared to those treated with the control or monotherapies (Figures 5e; S6b, Supporting Information). These findings suggest that the combination of Olaparib and LLY‐283 induces a robust anti‐tumor immune response by promoting the synergy between DC cells and CD8^+^ T cells.

To directly assess the role of dendritic cells (DCs) in mediating this immune activation, we performed DC depletion experiments in the EMT6 syngeneic breast cancer model. Mice were randomized into four groups: PBS control, DC depletion (anti‐CD11c + anti‐MHC‐II); Olaparib + LLY‐283, and Olaparib + LLY‐283 with DC depletion. As expected, Olaparib + LLY‐283 treatment significantly suppressed tumor growth compared to control. However, DC depletion alone had no effect, and importantly, DC‐depleted mice receiving the combination therapy lost the antitumor benefit (Figure 5f,g). These data demonstrate that DCs are indispensable for antigen presentation and T cell cross‐priming in our regimen, thereby substantiating the mechanistic requirement for DC‐mediated initiation of the CD8^+^ T cell‐driven antitumor immunity.

To further assess whether this reprogrammed TME translates into an immunotherapeutic effect, we evaluated the efficacy of the Combo therapy (Olaparib + PRMT5i + anti‐PD‐1 antibody) and compared the survival benefits with those of monotherapy or any combination of two agents. Strikingly, the Combo group demonstrated the highest efficacy with no observed toxicity, as indicated by stable body weight, unremarkable histology in major organs (H&E staining), and normal complete blood counts (Figures 5h; S7a–d, Table S9, Supporting Information). This remarkable efficacy of the three‐drug combination directly translated into the most substantial improvement in survival (Figure 5i). Consistently, scRNA‐seq analysis validated the activation of cytotoxic CD8^+^ T cells and DC cells in the Combo group (Figures 5j–l; S7e,f, Supporting Information). These findings suggest that the three‐drug combination therapy has the potential to induce a robust and durable antitumor immune response, which is crucial for achieving long‐term clinical benefits in TNBC patients.

## 3 Discussion

PRMT5 inhibitors such as JNJ‐64619178 and AZD3470 are under clinical evaluation for advanced solid tumors and hematologic malignancies (NCT03573310, NCT06130553). Despite this progress, intrinsic and acquired resistance to PRMT5 inhibition persists as a major therapeutic barrier, with only a subset of patients demonstrating durable responses.^[^ ^31^ ^]^ While biomarkers like MTAP loss or splicing deficiency signatures predict sensitivity in acute myeloid leukemia, these associations lack generalizability across cancers.^[^ ^32^ ^]^ Similarly, context‐specific markers‐including p53 mutations in B‐cell lymphoma, MYC overexpression in pancreatic adenocarcinoma, and the CLNS1A/RIOK1 ratio in glioblastoma‐show no prognostic value for PRMT5 inhibitor efficacy in triple‐negative breast cancer (TNBC).^[^ ^33^, ^34^ ^]^ This heterogeneity underscores the urgent need to define precise biomarkers to stratify patients most likely to benefit from PRMT5‐targeted therapies, particularly in TNBC.

By integrating selective PRMT5 inhibitors (GSK591, LLY‐283) with transcriptomic profiling, we identified basal interferon (IFN) signaling as a novel biomarker of PRMT5 inhibitor sensitivity in TNBC. Mechanistically, constitutive IFN pathway activation—driven by DNA damage response (DDR)‐mediated priming—creates a therapeutic vulnerability to PRMT5 loss, as validated in patient‐derived organoids. Strikingly, inducing DNA damage with olaparib in resistant models amplified IFN signaling and restored PRMT5 inhibitor sensitivity, suggesting a strategy to overcome resistance. This DDR‐IFN‐PRMT5 axis not only enhances tumor cell‐intrinsic apoptosis but also reprograms the immune microenvironment, synergizing with checkpoint blockade to sustain antitumor immunity.

These findings extend prior reports of PRMT5/PARP inhibitor synergy^[^ ^35^, ^36^ ^]^ by revealing IFN pathway activation as a predictive biomarker and immune‐modulating mechanism. While earlier studies emphasized PRMT5's role in impairing DNA repair, we demonstrate that preexisting IFN‐driven inflammation licenses dual DDR and immune vulnerability in TNBC. Our findings also extended the established role of PARP inhibitors in targeting DDR‐deficient tumors. While PARP inhibitors have been approved for homologous recombination (HR)‐deficient cancers such as those with BRCA1/2 mutations, treatment resistance due to reversion mutations or compensatory repair pathways remains a major challenge.^[^ ^37^ ^]^ PARP inhibitors induced DNA damage, which in turn activates the cGAS/STING pathway, triggering IFN signaling and enhancing tumor immunogenicity‐thus providing a mechanistic rationale for synergy with immune checkpoint blockade.^[^ ^38^, ^39^, ^40^, ^41^, ^42^ ^]^

To translate these findings, we propose a staged clinical validation strategy. First, retrospective analyses of archived biospecimens from completed PRMT5 or PARP inhibitor trials will be used to correlate baseline IFN signatures and DDR status with clinical outcomes. Second, we advocate designing prospective, biomarker‐driven early‐phase trials testing PRMT5 inhibitors combined with PARP inhibitors, with or without PD‐1 blockade, in TNBC, incorporating pre‐specified IFN/DDR stratification and comprehensive pharmacodynamic and toxicity monitoring. Key clinical readouts should include response rates in IFN‐high vs IFN‐low subgroups, duration of benefit, and safety endpoints to define tolerated schedules that preserve immune function.

We acknowledge limitations. Our mechanistic and efficacy data are principally preclinical; prospective clinical validation is required to establish the predictive value and utility of IFN signatures. Although we validated the combination across multiple organoid, PDX, and PDC models, further evaluation in larger and more genetically diverse clinical cohorts will be important to define the boundary conditions for efficacy (e.g., tumor intrinsic features, prior therapies, and tumor microenvironment composition). Finally, because combinations of DNA‐damaging agents and immune modulators may increase toxicities, careful dose‐finding and sequencing studies will be essential.

In summary, we reveal a therapeutically exploitable dependency in IFN‐primed TNBC tumors, wherein intrinsic DNA damage licenses sensitivity to PRMT5 inhibition while simultaneously enhancing immunogenicity. This dual mechanism provides a rationale for biomarker‐driven trials of PRMT5 inhibitors in TNBC and combination approaches to amplify durable clinical responses.

## 4 Experimental Section

### Animals

An experimental animal production license was obtained with the number: [SCXK (Zhejiang): 2019‐0002]. Five‐ to six‐week‐old female BALB/c (nude) mice were purchased from Laboratory Animal Center, Hangzhou Medical College, and housed in a specific pathogen‐free facility with free access to food and water, and a 12‐h light/dark cycle. To establish a tumor‐bearing mouse model, EMT6 (2×10^5^) cells were subcutaneously injected into immunocompetent BALB/c mice. Once tumors became palpable, mice were randomized and treated with vehicle, monotherapies (LLY‐283 at 50 mg kg^−1^ for 3 days on/4 days off from MedChemExpress, anti‐PD‐1 antibody at 200 µg every three days from BioCell, or Olaparib at 50 mg kg^−1^ daily via intraperitoneal injection from MedChemExpress), dual combinations of these drugs, or a triple combination of LLY‐283, anti‐PD‐1, and Olaparib. Tumor volumes were measured regularly, and survival curves were generated to assess treatment efficacy.

### Patients

The transcriptomes from patients with TNBC were collected from The Cancer Genome Atlas (TCGA).

### Cell Lines

TNBC cell lines were obtained from ATCC and cultured in DMEM (Gibco|Thermo Fisher Scientific, Waltham, MA, USA) or RPMI 1640 (Life Technologies, Carlsbad, CA, USA), supplemented with 10% fetal bovine serum (Merck, Rahway, NJ, USA) and 1% penicillin/streptomycin (Gibco) in a humidified atmosphere of 5% CO_2_ at 37 °C. Details of the cell lines used in this study are summarized in Table S1 (Supporting Information).

### Patient‐Derived Xenograft (PDX) Model

Nude mice transplanted with PDX tumors were randomly divided into four groups. Mice were administered normal saline, LLY‐283 (50 mg kg^−1^ via gavage, 3 days on and 4 days off), Olaparib (50 mg kg^−1^, once daily via intraperitoneal injection), or LLY‐283 + Olaparib for four weeks. PDX‐TNBC‐1 was established from a TNBC patient of Zhejiang Cancer Hospital, who experienced relapse following treatment with the AC‐T chemotherapy regimen (doxorubicin/cyclophosphamide followed by paclitaxel. The PDX‐TNBC‐2 and 3 were obtained from Nanchang Royo Biotech Co., Ltd. (Permit No. RYE 1551, and No. RYE 598).

### Organoids

Human tumors were collected from TNBC patients with informed consent, following the protocol approved by Zhejiang Cancer Hospital. Organoids were derived from PDX tumors (DCBXTO.58, DCBPTO.19, and DCBXTO.28) or human tumors (DCBPTO.66). Each model name follows a standardized six‐character code. DC: Dave Ceson Laboratory; B: Breast cancer; X or P: Source of the tumor (X indicates patient‐derived xenograft; P indicates direct patient biopsy); T: Tumor; O: Organoid. Accordingly, DCBXTO denotes organoids established from a Dave Ceson lab‐generated Breast cancer Xenograft Tumor as an Organoid, whereas DCBPTO indicates organoids derived directly from a Patient Tumor Organoid. Detailed metadata for each model is also available via the CancerModels.Org database.

Tumor tissue was minced and digested in 5–10 mL of advanced DMEM containing 1× GlutaMAX, 10 mm HEPES, 1× antibiotic‐antimycotic (AdDF^+++^), and 250–500 µg mL^−1^ Liberase TH for 45 min at 37 °C with gentle rocking. Tissue was filtered through a 100 µm cell strainer and centrifuged at 400 g for 10 min at 4 °C. Pellets were washed once with AdDF^+++^, centrifuged, and treated with red cell lysis buffer Hybri‐Max for 5 min on ice before cell counting. Organoids were cultured as previously described.^[^ ^15^ ^]^ PDX‐derived organoids were evaluated for human and mouse cell content by flow cytometry to ensure purity. Additional quality control steps included short tandem repeat analysis to confirm organoid matching to the tumor of origin and mycoplasma testing.

### Organoid Drug Treatment

For drug treatment, organoids were dissociated into single cells and seeded in 48‐well plates in basement membrane extracts (BME) at a density of 2000 cells per well. After BME solidified, the organoid/BME domes were overlaid with 475 µL of medium with or without drug. Fresh medium and drugs were applied every 5 days. A well containing BME only was used as a negative control. The cells were cultured for 12–21 days, depending on the growth rate of the model, until the untreated controls formed organoids of > 50 mm in diameter. After removing the medium, the organoids were incubated with 1× PrestoBlue HS Reagent (Thermo Fisher) in breast organoid media^[^ ^15^ ^]^ overnight at 37 °C. On the following day, aliquots of the medium were transferred to a 384‐well plate, and fluorescent intensity was measured at 560/590 nm excitation/emission wavelength using a CLARIOstar Plus microplate reader. The medium‐only control was used for background correction. Cell viability for each organoid model was normalized to the corresponding BME‐only control. Three independent assays were performed for each organoid model.

### Patient‐Derived Cell Models

Tumors were collected from 4 breast cancer patients who underwent surgical resection at Zhejiang Cancer Hospital in 2025 (Table S10, Supporting Information). Samples were washed with pre‐cold PBS and then cut into 0.5 mm^2^ pieces and dissociated into cell suspensions using dissociation solvent (0.35% collagenase IV5, 2 mg mL^−1^ papain, 120 Units mL^−1^ DNase I). The cell suspensions were collected, filtered through a 40 µm cell strainer, and washed twice with PBS. Subsequently, the cells were cultured with Dulbecco's modified Eagle's medium (DMEM; YESEN, 41401ES) or RPMI 1640 (YESEN, 41402ES) with 10% FBS (Sunrise, SR100180.03) and 10% penicillin/streptomycin (Biosharp, BL505A), and incubated at 37 °C in an incubator containing 5% CO_2_. After 24 h of culture, cells were plated at 2000 cells per well in 384‐well plates with 10 µm olaparib, 10 µm PRMT5 inhibitor, and their combination for 5 days of treatment. Imaged and analyzed with the Incucyte SX5 live cell imaging device (Sartorius, Germany). The experiments were approved by the Ethics Committee of Zhejiang Cancer Hospital (Approval number: IRB‐2021‐410).

### Bioinformatics Analysis

Data collected from compound activity and cell viability assays were processed using an IncuCyte ZOOM live‐cell analysis system (Essen492 Biosciences). Data were normalized to the DMSO control and analyzed using R (v3.5.1) with Bioconductor (v.3.14). The half‐maximal inhibitory concentration (IC_50_) and the area above the curve (AAC) were calculated using the PharmacoGx R package (v. 2.6.0). The transcriptomes of TNBC cell lines were acquired through RNA‐seq and processed using the Kallisto pipeline.^[^ ^43^ ^]^ Genes were ranked according to Pearson correlation coefficients between AAC and individual gene expression levels. Hallmark gene sets were downloaded from MsigDB (https://www.gsea‐msigdb.org/gsea/msigdb/).^[^ ^44^ ^]^ GSEA plots were generated using the piano R package and the fgsea R package.^[^ ^45^, ^46^ ^]^

### IFN Signature Scoring

The IFN activation signature was derived by intersecting differentially expressed genes in sensitive cell lines and patient‐derived organoids (PDOs) with the MSigDB hallmark “Interferon Alpha Response” and “Interferon Gamma Response” gene sets, resulting in a non‐redundant gene list.

### Cell Viability Assay

Cells were seeded in 384‐well plates and treated with various doses of PRMTi for 5 days. After treatment, cell viability assay reagent CCK8 (Biosharp, BS350B) was added to each well to reach a 10% final concentration. The absorbance was measured using a multimode microplate reader (TECAN, Spark).

### Immunofluorescence Staining

Cells were cultured on poly‐L‐ornithine‐pretreated coverslips in 12‐well plates and treated with LLY‐283 or DMSO for 5 days. Following treatment, cells were washed with PBS and fixed in 4% formaldehyde for 15 min at room temperature. Subsequently, Cells were permeabilized using 0.5% (V/V) Triton X‐100 for 10 min and then blocked with 5% bovine serum albumin in PBS for 1 h at room temperature, followed by incubation with anti‐γ‐H2AX (1:500, #9718S, CST) overnight at 4 °C. After incubation with the secondary antibody (1:1000; Cell Signaling Technology) for 1 h in dark at room temperature, coverslips were washed with PBS and mounted using DAPI‐containing mounting medium (Invitrogen, Waltham, MA, USA). Images were captured using an A1 HD25 Single‐Photon confocal microscope and analyzed by NIS‐Elements (v5.21.03).

Tumor‐infiltrating CD8+ T cells in paraffin‐embedded tumor tissue sections from BALB/c mice bearing EMT6 tumors. The mice were treated with LLY‐283 (50 mg kg^−1^, 3 days on, 4 days off), Olaparib (50 mg kg^−1^, daily), or a combination of both. Tissue sections were deparaffinized in xylene and rehydrated through graded ethanol. Antigen retrieval was performed in EDTA buffer, followed by peroxidase blocking with 3% H_2_O_2_ and serum blocking with 3% BSA. The staining process specifically targeted CD8+ T cells using a CD8 primary antibody (CST #98941S; 1:400, 4 °C overnight), treated with HRP‐conjugated universal secondary antibody (Hangzhou Hawk, HKI0005), amplified with Flare520 fluorophore (Hangzhou Hawk, HKI0014), and counterstained with DAPI, with PBS washes between steps. For quantification, images were captured using a Nikon ECLIPSE C1 fluorescence microscope with a scale bar of 50 µm. Three random fields per section were quantified by manual counting, normalized to field area (cells mm^−2^), with *n* = 4 biological replicates. Data are presented as mean ± standard deviation, and statistical analysis was performed using one‐way ANOVA with Dunnett's test for multiple comparisons to assess differences across treatment groups.

### Quantitative Real‐Time PCR (qRT‐PCR)

Total RNA was isolated using the Total RNA Extraction Reagent kit (Vazyme, Jiangsu, China), and cDNA synthesis was performed with the iScript gDNA Clear cDNA synthesis kit (Yeasen, Shanghai, China) following the manufacturer's protocol. PCR was performed using qPCR SYBR green master mix (Vazyme, Jiangsu, China) on a LightCycler 96 real‐time PCR System device. Primers were summarized in Table S11 (Supporting Information). Relative gene expression was calculated using the 2^−ΔΔCT^ method.

### Western Blot Analysis

Cells and tumor tissue samples were lysed as previously described.^[^ ^47^ ^]^ A total of 20–100 µg of proteins were separated in 4–12% Bis‐Tris protein gels (Invitrogen) and transferred to nitrocellulose membranes (Bio‐Rad). Primary antibodies included anti‐PRMT5 (CST, 79998S), anti‐SDMA (CST, 13222S), anti‐γ‐H2AX (CST, 9718S), anti‐GAPDH (HUABIO, ET1602‐4), anti‐TBK1(CST, 38066S), anti‐p‐TBK1(CST, 5483S), anti‐STING (CST, 50494S), anti‐IRF3 (HUABIO, ET1612‐14), anti‐p‐IRF3 (HUABIO, ET1608‐22), anti‐GINS4 (proteintech, 13315‐1‐AP). Images were acquired using an Odyssey scanner (LiCor, Lincoln, NE, USA) or Amershan ImageQuant 80 and analyzed using ImageQuant TL (v8.2.0) and ImageJ (v1.53a).

### siRNA Transfection

Cells were seeded overnight in 6‐well plates at a density of 2.5 × 10⁵ cells per well. Transfection complexes were prepared by combining siRNA (final concentration 50 nm, see Table S12, Supporting Information) and Transfection Reagent at the manufacturer‐recommended ratio in serum‐free medium, followed by incubation at room temperature for 5 min. Complexes were added dropwise to the cells. Gene expression analysis was performed 48 h post‐transfection.

### Flow Cytometry

For tumor tissue, at the end of the animal experiment, tumors were collected, chopped, and digested for 30 min at 37 °C using tumor tissue dissociation solution (Orgarid, SD004) according to the manufacturer's protocol to obtain a single cell suspension. The suspension was then passed through a 40 µm cell strainer and centrifuged. Cell pellets were obtained, washed, and resuspended in PBS. Cells were incubated with live/dead cell viability reagent for 30 min on ice. Then, cells were washed twice with volumes of neutralizing PBS containing 2% FBS. For surface staining, cells were incubated with the antibodies diluted in PBS at 4 °C for 30 min. After staining, cells were washed and resuspended in PBS. Samples were analyzed on a CytoFLEX LX (Beckman). Compensation and gating were performed on CytExpert software (v2.4). The antibodies used are detailed in Table S13 (Supporting Information).

### RNA Sequencing

RNA was extracted from TNBC cell lines and organoids using TRIzol (Thermo Fisher Scientific, cat. 15596018). RNA quantity was assessed with a NanoDrop ND‐1000 (NanoDrop, Wilmington, DE, USA), and RNA integrity was evaluated using a Bioanalyzer 2100 (Agilent, Santa Clara, CA, USA). mRNA was purified using oligo(dT) beads (Dynabeads Oligo (dT), cat. 25–61005, Thermo Fisher Scientific). Purified mRNA was fragmented, and RNA libraries were constructed following the manufacturer's instructions for the NEBNext Ultra Directional RNA Library Prep Kit (NEB, cat. E7760). Paired‐end sequencing was performed on an Illumina NovaSeq 6000 platform. FASTQ files were aligned to the human genome (GRCh38) using Bowtie2 (v2.2.5). SAM files were converted to BAM files with SAMtools (v1.10). The Repenrich2_subset function was used to generate files for uniquely and multimapped reads, which were analyzed to estimate repetitive element expression using the Repenrich2 function. Differential expression was analyzed using DESeq2 (v1.34.0) with FDR correction (see subhead Quantification and Statistical Analysis in Experimental Section).

### Whole–Exome Sequencing

DNA was extracted from the patient‐derived tumor xenograft and quantified using Qubit (1×dsDNA HS Assay kit, YEASEN, cat. 12642ES76). For whole‐exome sequencing, libraries were prepared using the Twist Library Preparation EF Kit 2.0 (Twist Bioscience, cat. 104207) followed by target enrichment with the Twist Exome 2.0 Plus Panel (Twist Bioscience, cat. 105036). Sequencing was performed on the Illumina NovaSeq X Plus platform with 150‐base pair paired‐end reads (PE150). FASTQ files were trimmed with Trim Galore (v0.6.7, Q20) and aligned to GRCh38 using BWA‐MEM (v0.7.17). PCR duplicates were removed (GATK v4.2.6.1), and variants were called with GATK HaplotypeCaller, filtered (QUAL < 30, QD < 2, FS > 60, SOR > 3, MQ < 40), and annotated using ANNOVAR (RefSeq).

### Splicing Analysis

rMATS (version 4.1.1) was used to identify alternative splicing events and analyze differential alternative splicing events between samples. AS events were identified with a false discovery rate (FDR) < 0.05 in a comparison as significant AS events.

### Single‐Cell RNA‐Seq Data Analysis

After treatment, tumors were collected from BALB/c mice. Tumors displaying no necrosis and ulcers in the area from three mice were pooled together as one sample. Tumors were dissociated into single‐cell suspensions for sequencing using a tumor dissociation kit. At least 5000 cells in each group were used for scRNA‐seq library preparation using the Single Cell 3′ Reagent Kits (10× Genomics). The libraries were processed on an Illumina NovaSeq 6000 system. FASTQ files were converted into count matrices by Cell Ranger Software (v7.0.0), with the Mus musculus genome mm10 serving as the reference genome. A gene expression matrix was generated using the Seurat package (v4.3.0). The t‐Distributed Stochastic Neighbor Embedding (t‐SNE) method was used for visualization. The K‐nearest neighbor graph was constructed using the *FindNeighbors* function. The clustering was performed with the *FindClusters* function using a resolution of 1.3. Cell‐type annotation was executed through manual curation, selecting markers from relevant studies pertaining to the same cell populations, facilitated by the *AddModuleScore* function. Known markers used for cell‐type annotation are provided in Table S14 (Supporting Information). For more detailed cell type annotation, *Epcam*, *Krt18*, and *Krt8* genes were used to distinguish tumor cells, while the remaining cells were re‐clustered into eight groups. Markers used for cell‐type annotation can be found in Table S15 (Supporting Information).

### Quantification and Statistical Analysis

Data were presented as the mean ± standard deviation and analyzed using two‐way ANOVA followed by Dunnett's test for multiple comparisons. A *p*‐value less than 0.05 was considered statistically significant. Tumor growth curves in BALB/c mice were analyzed using a two‐sided unpaired t‐test to compare treatment groups at specific time points. Tumor volumes were measured using length × width^2^ × 0.5. Survival data were evaluated using Kaplan–Meier curves and log‐rank tests. Transcriptomic analyses used Pearson correlation coefficients to rank genes by AAC correlation, with gene set enrichment analysis (GSEA) performed using the clusterProfiler R packages. Western blot densitometry and qRT‐PCR (2‐ΔΔCT) data were compared using a two‐sided unpaired t‐test. Single‐cell RNA‐seq differential expression was analyzed using Wilcoxon rank‐sum tests in the Seurat package (v4.3.0). Drug synergy was assessed using the Bliss independence model via SynergyFinder (v3.16.0). Specific statistical tests for each experiment are detailed in the corresponding figure legends.

### Bliss Synergy Scoring

The Bliss independence model is a reference model for evaluating the combination effect of drugs. The expected combined effect (Ee) is calculated using the following equation:

(1) Ee=Ea+Eb−Ea×Eb

For each concentration pair of the two drugs, the Bliss synergy score is computed as:

(2) BlissScore=Eo−Ee

Where Ee is the expected combined effect. Ea and Eb are the effects of Drug a and Drug b, respectively. Eo is the observed combined effect. If the observed combined effect Eo exceeds Ee, the combination is considered synergistic; otherwise, it may be additive or antagonistic.

The Bliss synergy scores were calculated using the CalculateSynergy function from the SynergyFinder (v3.16.0) R package. The synergy scores were subsequently visualized using the Plot2DrugHeatmap and PlotSynergy functions. Unless otherwise specified, all calculations were performed using the default parameter settings of the package.″

### Zero Interaction Potency (zip) Scoring

The analysis of synergistic effects was performed using the SynergyFinder online analysis platform, employing the Bliss model and ZIP model.

### Statistical Analysis

Prism software (GraphPad) was used for statistical analysis and graphical representation of the data. Statistical significances were tested using an unpaired Student's t‐test. All tests were considered statistically significant when *P* < 0.05.

## Conflict of Interest

The authors declare no conflict of interest.

## Author Contributions

Z.W.Z., S.Y.Z., and L.G. contributed equally to this work. Q.W. supervised the research. Z.W.Z., S.Y.Z., X.J.W., and Y.S.J.planned the experiments, designed and prepared figures, and performed the majority of the assays. Y.S.J., J.H., and S.Y.Z. performed the RNA‐seq analysis. L.G. performed the Single‐cell RNA‐seq analysis. J.Q.T. conducted qRT‐PCR, cell growth, western blotting, genetic knockdown studies, and dsRNA staining assays. S.Y.Z., Y.C.P. performed the organoid assays, flow cytometry, and mouse studies. Z.W.Z., Y.W., and Q.W. wrote the manuscript. All authors assisted in writing the manuscript, reviewed the final version, and approved the content and submission.

## Supporting information



Supporting Information

Supplemental Table 1

Supplemental Table 2

Supplemental Table 3

Supplemental Table 4

Supplemental Table 5

Supplemental Table 6

Supplemental Table 7

Supplemental Table 8

Supplemental Table 9

Supplemental Table 10

Supplemental Table 11

Supplemental Table 12

Supplemental Table 13

Supplemental Table 14

Supplemental Table 15

## Data Availability

The data underlying this article are available in the article and in its online supplementary material. And the associated data has been deposited to GEO with code: GSE292508.
