# Supplementary material for: Interferon‐Driven Biomarkers and Synergistic Therapy for PRMT5 Inhibition in Triple‐Negative Breast Cancer
Source: Adv Sci (Weinh). 2025 Nov 19;13(6):e05787. doi: 10.1002/advs.202505787 (PMC12866763; doi:10.1002/advs.202505787)
Supplement: Supplementary file 1 — Supporting Information [file ADVS-13-e05787-s003.docx]

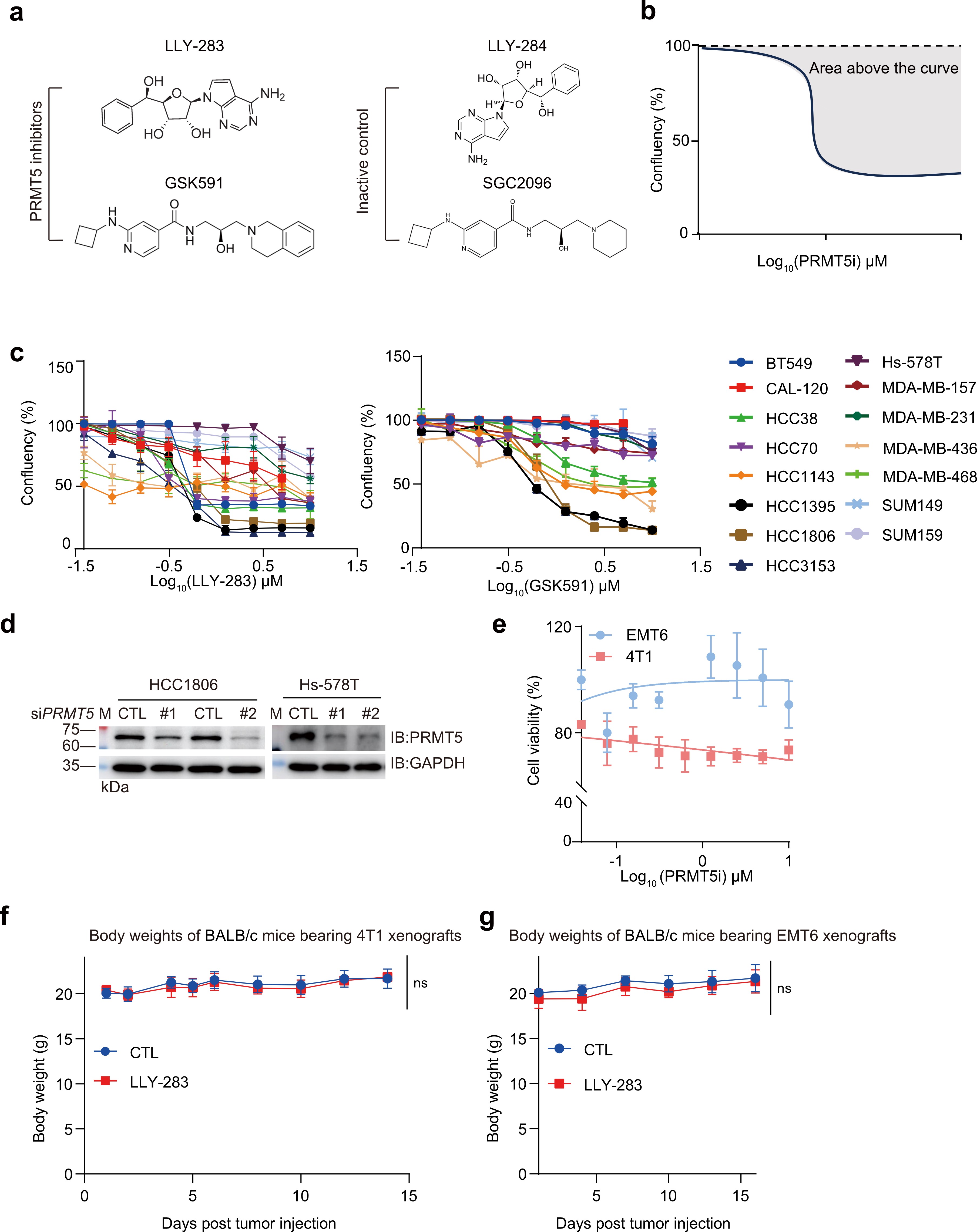
Supplementary Fig. 1: Heterogeneous response to PRMT5 inhibition in TNBC.

**a,** Chemical structures of PRMT5 inhibitors LLY-283 and GSK591, alongside the inactivated controls LLY-284 and SGC2096. **b,** Schematic diagram illustrates AAC (area of above curve) used for sensitivity analysis. **c,** Cell proliferation curves of TNBC cell lines treated with LLY-283 (left panel) or GSK591 (right panel) for 5 days. Data are shown as mean ± s.d., *n* = 4. **d**, Immunoblots of PRMT5 of siRNA knockdown of *PRMT5* or control in HCC1806 (left panel) and Hs-578T (right panel) cells. Data are representative of independent experiments. **e**, Growth curves of 4T1 and EMT6 cells treated with LLY-283 for 5 days at indicated concentrations. Data are shown as mean ± s.d., *n* = 3. **f-g**, The body weight of BALB/c mice bearing 4T1 xenografts (f) and EMT6 xenografts (**g**). Data are presented as mean ± s.d., *n* = 3, two-sided unpaired *t*-test.


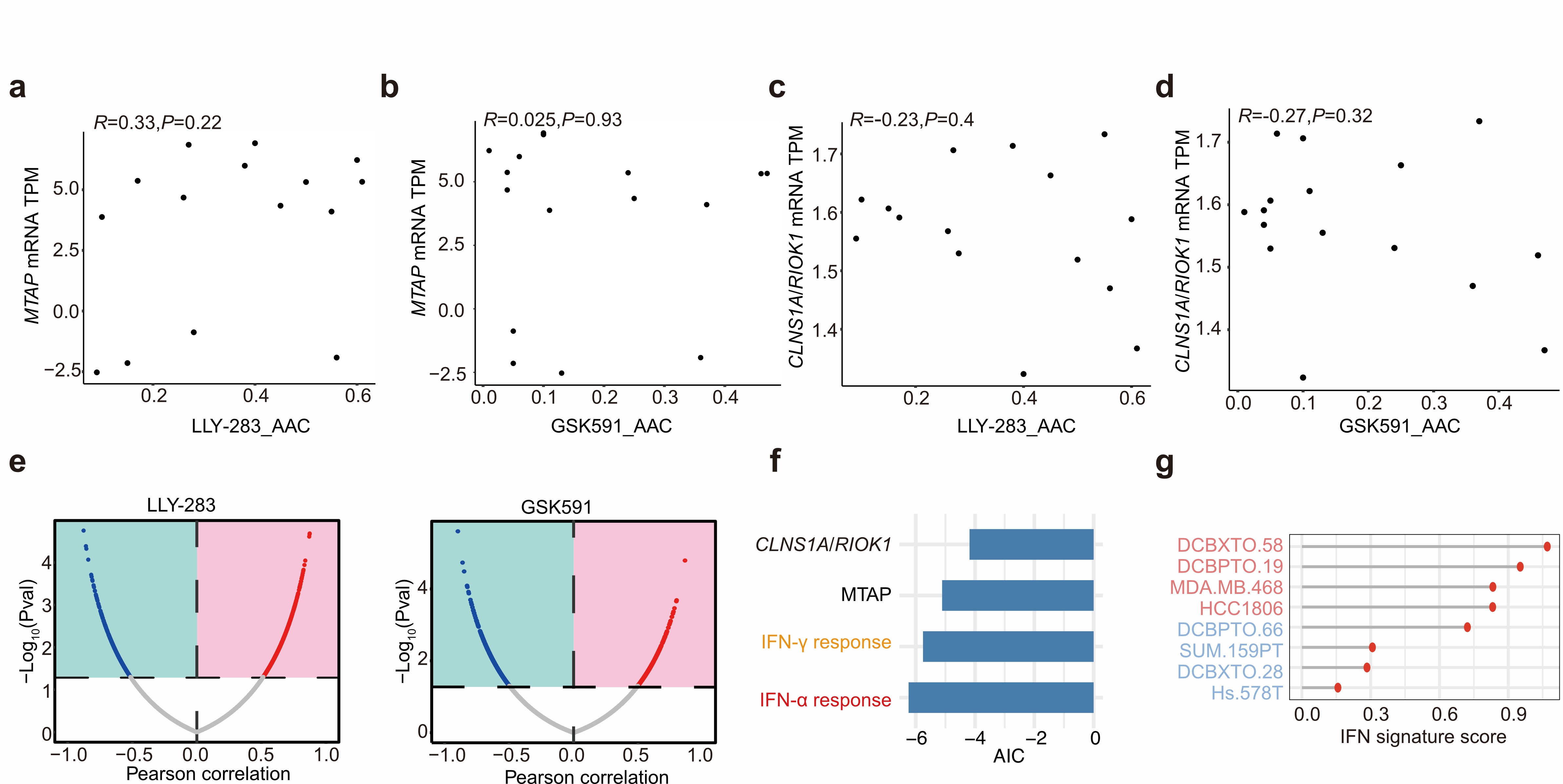


Supplementary Fig. 2: Basal interferon response correlates with TNBC sensitivity to PRMT5 inhibition.

**a-b,** Correlation of *MTAP* mRNA expression with LLY-283 (**a**) or GSK591 (**b**) sensitivity. **c-d,** Correlation of *CLNS1A*/*RIOK1* ratio with LLY-283 (**c**) or GSK591 (**d**) sensitivity. **e**, Pearson correlation analysis of gene-expression profiles and sensitivity to LLY-283 (left panel) and GSK591 (right panel) across TNBC cell lines. Red indicates upregulated genes associated with increased sensitivity. Blue indicates negative correlations associated with resistance. **f**, Akaike Information Criterion (AIC) values for each candidate biomarker. **G,** Lollipop plot of interferon-signature scores across cell lines and PDOs models.


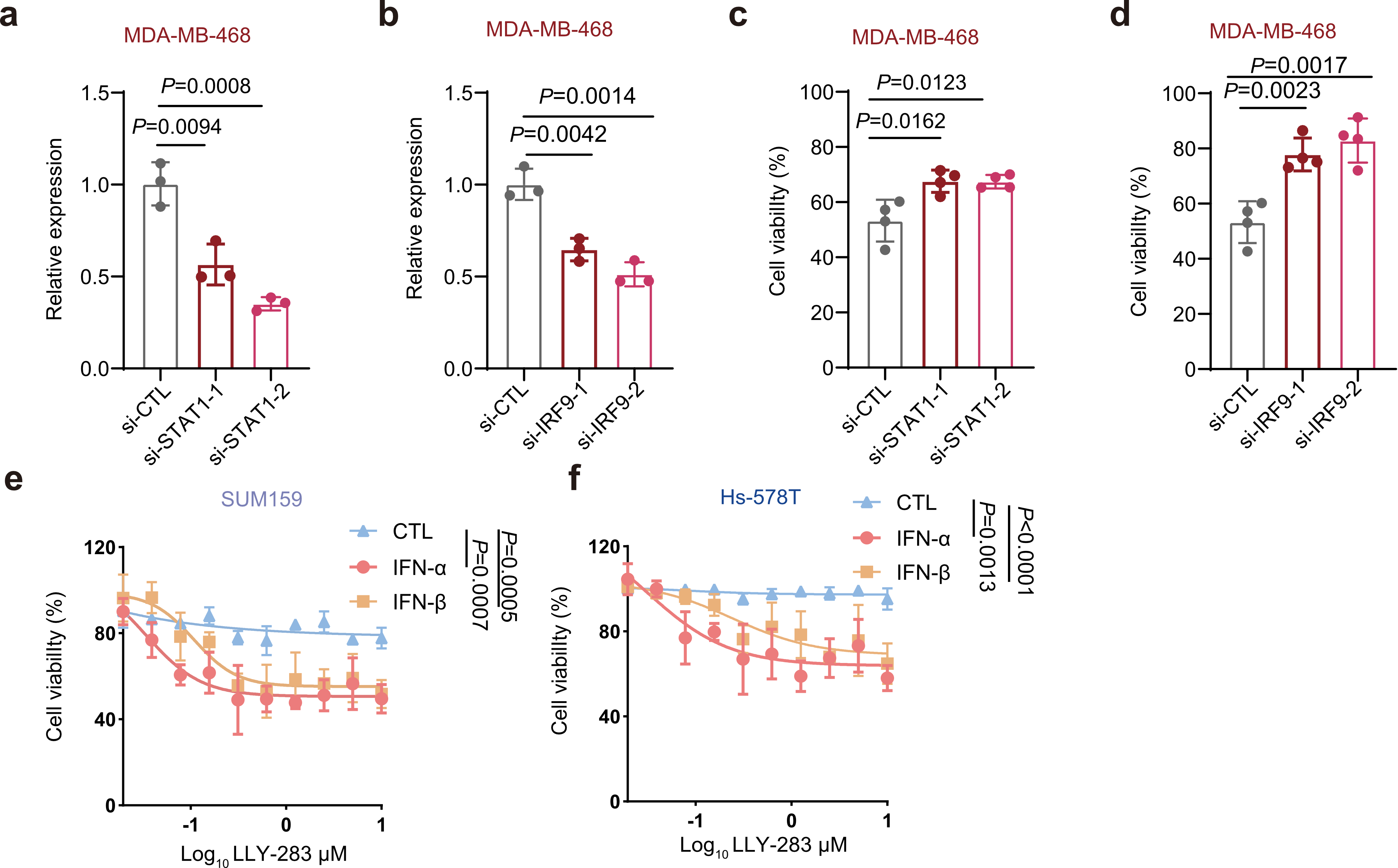
Supplementary Fig. 3: IFN signaling in PRMT5i sensitivity.

**a,** *STAT1* expression of MDA-MB-468 cells transfected with indicated siRNAs, Data are presented as mean ± s.d., *n* = 3, two-sided unpaired *t*-test. **b,** *IRF9* expression of MDA-MB-468 cells transfected with indicated siRNA, Data are presented as mean ± s.d., *n* = 3, two-sided unpaired *t*-test. **c,** Cell viability of MDA-MB-468 cells and their *STAT1*-knockdown derivatives treated with LLY-283, Data are presented as mean ± s.d., *n* = 4, two-sided unpaired *t*-test. **d,** Cell viability of MDA-MB-468 cells and their *IRF9*-knockdown derivatives were treated with LLY-283, Data are presented as mean ± s.d., *n* = 4, two-sided unpaired *t*-test. **e-f,** Cell viability of SUM159 (**e**) and Hs-578T cells (**f**) treated with IFN-α or IFN-β was assessed following treatment with gradient-diluted LLY-283, Data are presented as mean ± s.d., *n* = 4, two-sided unpaired *t*-test.


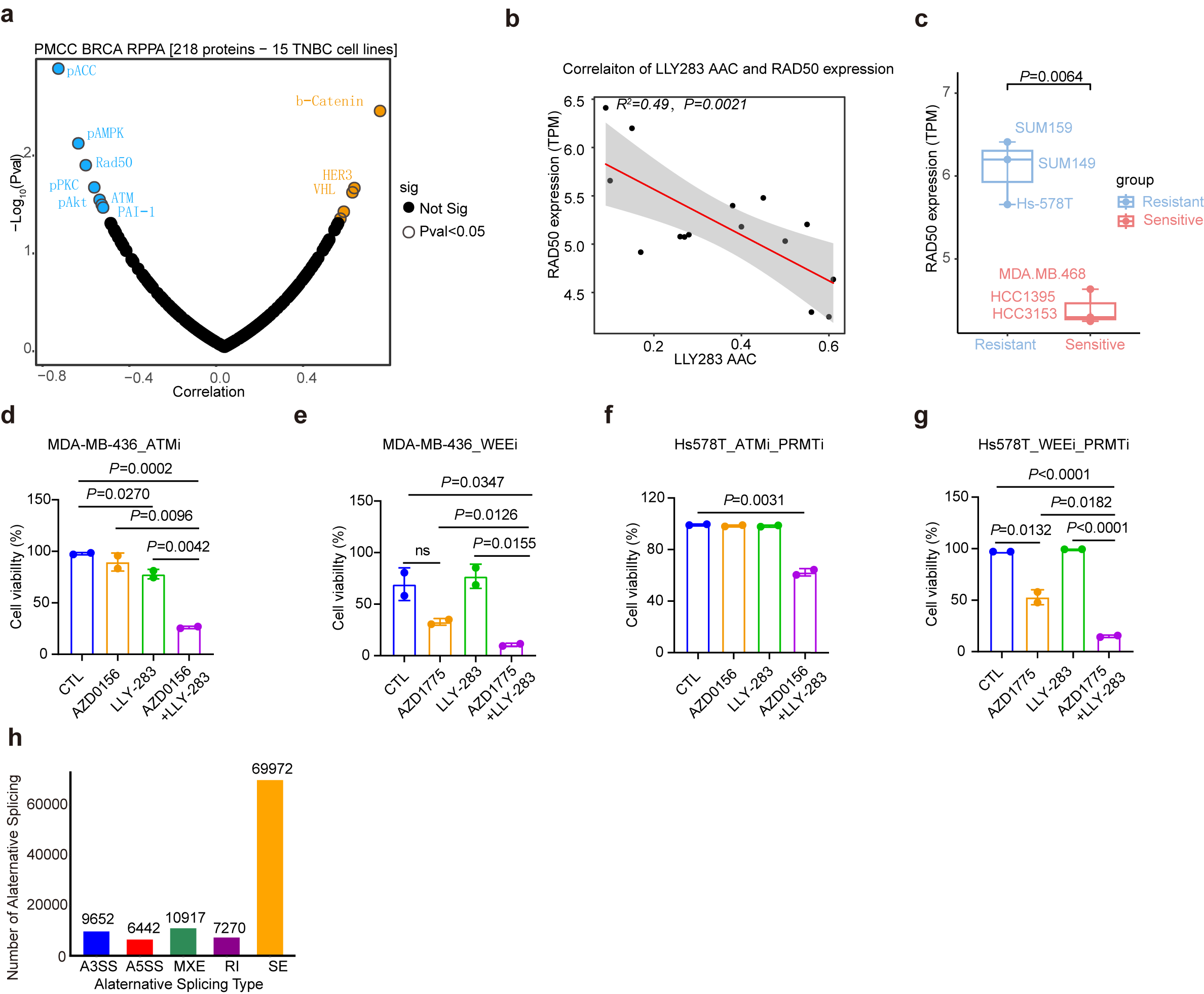


Supplementary Fig. 4: DNA damage response drives PRMT5i resistance in TNBC models.

**a,** Volcano plot showing proteins whose abundance correlates with sensitivity to the PRMT5 inhibitor LLY-283. Log_2_ fold change values compare protein levels in sensitive lines (red, right) versus in resistant lines (blue, left). Data were analyzed by unpaired two-tailed Student’s *t*-test for multiple comparisons. **b,** Correlation of *RAD50* mRNA expression with LLY-283 sensitivity. **c,** Boxplot indicated *RAD50* mRNA expression in sensitive cells (red, right) and resistance cells (blue, left). **d,** Cell viability of MDA-MB-436 cells wer**e** treated with AZD0156, LLY-283, or their combination. **e,** Cell viability of MDA-MB-436 cells were treated with AZD1175, LLY-283, or their combination. **f,** Cell viability of Hs-578T cells were treated with AZD0156, LLY-283, or their combination. **g,** Cell viability of Hs-578T cells were treated with AZD1175, LLY-283, or their combination. Data are presented as mean ± s.d., *n* = 2, statistical significance was evaluated using the two-tailed Student’s *t*-test. **h,** Bar plot showing the number of genes with alternative splicing (AS) events after interferon alpha treatment. A3SS (alternative 3’ splice site), A5SS (alternative 5’ splice site), MXE (mutually exclusive exon), RI (retained intron), and SE (skipped exon).


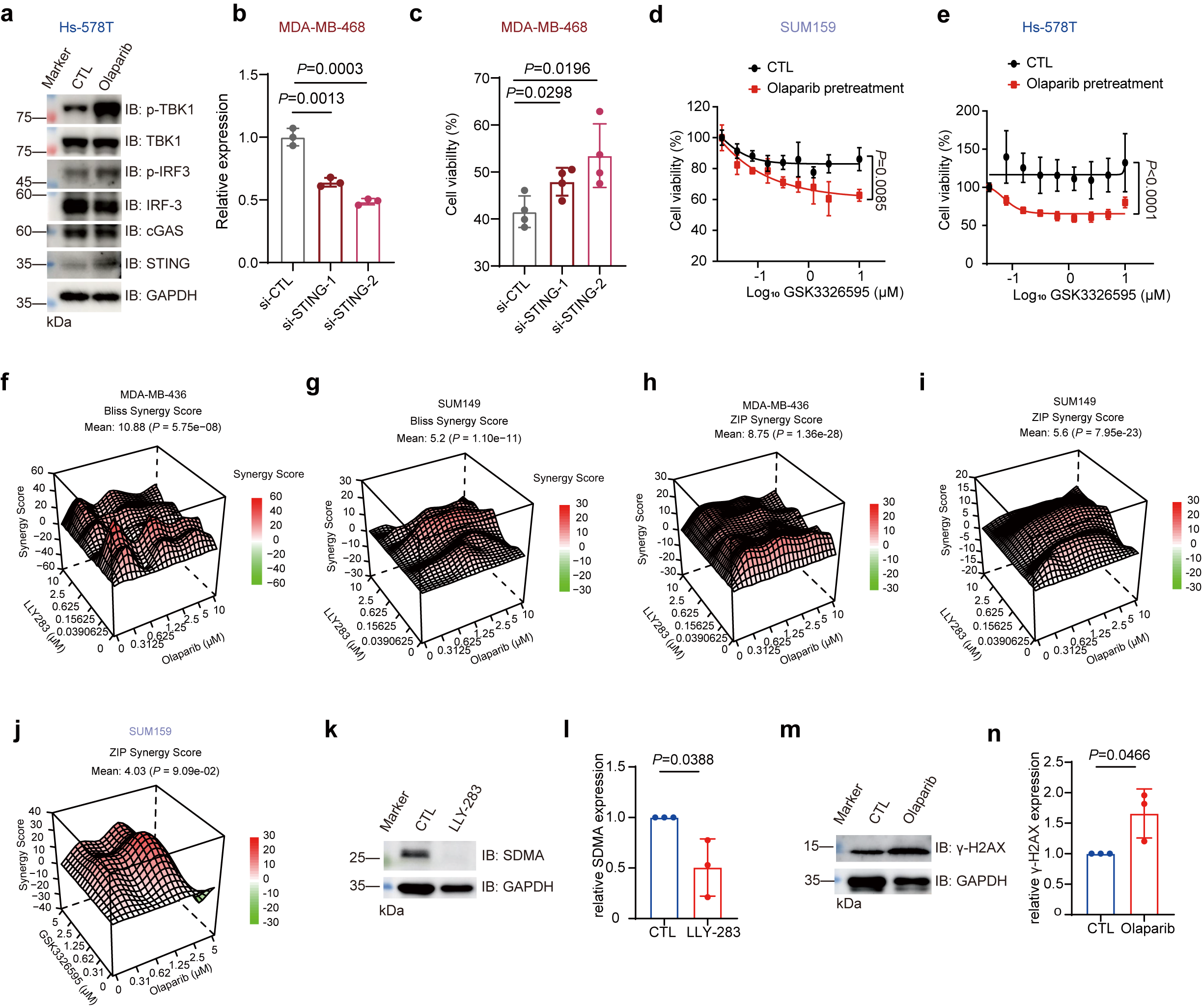


Supplementary Fig. 5: Synergistic sensitization of TNBC to PRMT5 inhibition by Olaparib.

**a,** Western blot analysis of pTBK1, TBK1, pIRF3, IRF3, cGAS, and STING expression of Olaparib treated Hs-578T cells and GAPDH as a loading control. **b,** qPCR quantification of *STING* mRNA expression in MDA-MB-468 cells transfected with control siRNA (siCTL) or siRNA targeting *STING1* (siSTING1), Data are shown as mean ± s.d., *n* = 3, two-sided unpaired *t*-test. **c,** Cell viability of MDA-MB-468 cells and their *STING*-knockdown derivatives treated with LLY-283, Data are shown as mean ± s.d., *n* = 3, two-tailed Student’s *t*-test. **d-e,** Cell viability curves of SUM159 (**d**) and Hs-578T (**e**) pretreated with Olaparib (3 µM, 3 days), followed by exposure to different concentrations of GSK3326595 for an additional 5 days. Data are shown as mean ± s.d., *n* = 3, two-sided unpaired *t*-test. **f-g**, Bliss Synergy Score for Olaparib + PRMT5 inhibitor combination in MDA-MB-436 (**f**) and SUM149 (**g**). **h**-**j**, Zip Synergy Score for Olaparib + PRMT5 inhibitor combination in MDA-MB-436 (**h**), SUM149 (**i**) and SUM159 (**j**) cells. **k,** Representative immunoblots of SDMA expression of tumor tissue from mice treated with control (CTL) or LLY-283. **l,** Quantification of SDMA band intensities from (**k**), Data are shown as mean ± s.d., *n*= 3, two-sided unpaired *t*-test. **m,** Representative immunoblots of γ-H2AX protein expression of tumor tissue from mice treated with control or Olaparib. **n,** Quantification of γ-H2AX band intensities from (**m**), Data are shown as mean ± s.d., *n* = 3, two-sided unpaired *t*-test.


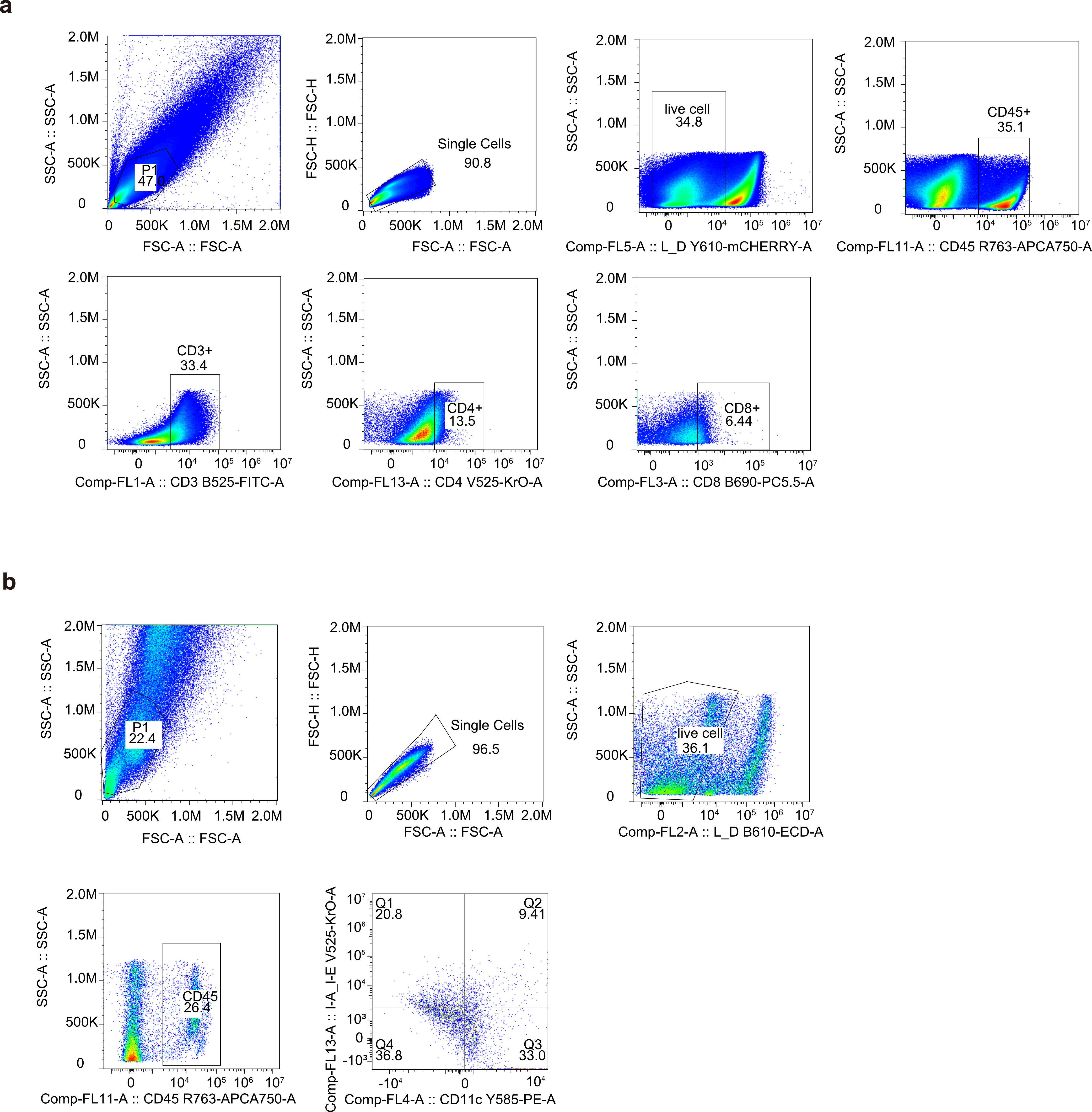


Supplementary Fig. 6: Flow cytometry gating plots of CD8^+^ T cells and dendritic cells.

**a**, Flow cytometry gating plots for quantifying CD8^+^ cells in CD45^+^ cells. **b**, Flow cytometry gating plots for quantifying MHC-class-Ⅱ^+^, CD11c^+^ cells in CD45^+^ cells.


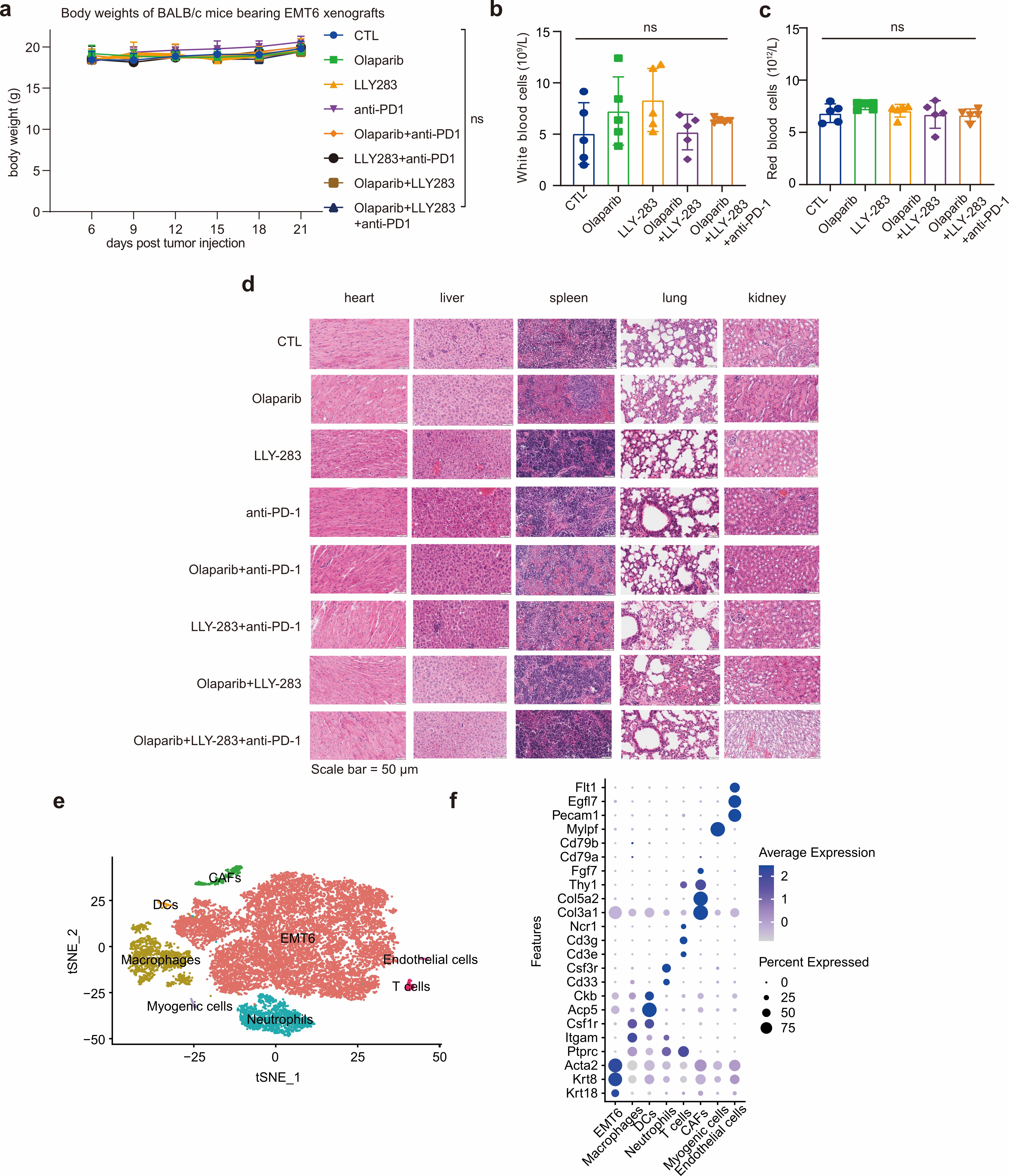


Supplementary Fig. 7: Olaparib enhances the efficacy of PRMT5 inhibition combined with anti-PD-1 immunotherapy.

**a-c**, Body weight (**a**), red blood cell counts (**b**) and white blood cell counts (**c**) of BALB/c mice bearing EMT6 xenografts were treated with LLY-283 (50 mg/kg, 3-day on and 4-day off), Olaparib (50 mg/kg, every day) and anti-PD-1 (200µg/mouse), alone or in combination. Data are shown as mean ± s.d., *n* = 5 or 8 per group, two-sided unpaired *t*-test. **d**, HE staining of the heart, liver, spleen, lung, and kidney tissue were performed. Scale bar, 50 µm. **e**, t-SNE plot of cell clusters of EMT6 tumors from two-drug (Olaparib and LLY-283) combination and three-drug (Olaparib, LLY-283 and anti-PD-1) combination groups. **f**, Dotplot of canonical marker genes used to annotate each cell cluster across all single-cell transcriptomes.
